# A Versatile Microfluidic Extrusion‐Based Hydrogel Platform for Self‐Organization and Long‐Term Maintenance of Engineered 3D Lymphatic Endothelium

**DOI:** 10.1002/adhm.71307

**Published:** 2026-06-03

**Authors:** Elsa Mazari‐Arrighi, Adeline Boyreau, Laura Chaillot, Wilfried Souleyreau, Laetitia Andrique, Pierre‐Olivier Guichet, Thomas Mathivet, Andreas Bikfalvi, Pierre Nassoy

**Affiliations:** ^1^ LP2N Laboratoire Photonique Numérique et Nanosciences Université de Bordeaux Talence France; ^2^ Institut d'Optique Graduate School CNRS UMR 5298 Talence France; ^3^ BRIC INSERM U1312 Université de Bordeaux Pessac France; ^4^ VoxCell 3D Facility UAR TBMcore CNRS 3427 INSERM US05 Université de Bordeaux Bordeaux France; ^5^ Université de Poitiers CHU Poitiers ProDiCeT Poitiers France

**Keywords:** 3D endothelium, extracellular matrix, lymphatic vessels, microfluidic, primary human lymphatic endothelial cells

## Abstract

Lymphatic endothelium is essential for interstitial fluid drainage, immune surveillance, and macromolecular transport. However, in vitro models that capture three‐dimensional (3D) architecture, lineage stability, and long‐term barrier properties remain limited. Here, we present a microfluidic extrusion‐based platform that generates size‐controlled 3D lymphatic endothelium from primary human lymphatic endothelial cells. Coaxial extrusion of an alginate shell around a hydrogel core yields tubular constructs whose inner diameter can be tuned from ∼50 to ∼300 µm by adjusting flow rates. We identified a four‐component hydrogel that supports self‐assembly of lymphatic endothelial cells into lumen‐forming monolayers within 1 week. These engineered tubes maintain viability, lymphatic marker expression, and selective macromolecular permeability for at least 30 days under static culture. Over time, the cells remodel the core into a stratified wall that includes a fibronectin‐rich perivascular zone. RNA sequencing shows that 3D lymphatic tubes are enriched in markers for lymphatic morphogenesis, matrix organization, and maturation compared with two‐dimensional monolayers. In parallel, the same matrix supports vascular endothelial cells that form long‐lived 3D tubes with key lineage‐specific transcriptional and barrier properties. Altogether, this platform provides a robust and tunable system for modeling lymphatic endothelium and dissecting structure–function relationships in engineered 3D microvessels.

## Graphical abstract

Microfluidic coaxial extrusion generates size‐controlled 3D lymphatic tubes from primary human dermal lymphatic endothelial cells in a defined four‐component matrix. These engineered vessels self‐organize into stable lymphatic endothelium, maintain selective macromolecular permeability for 30 days, and enable direct comparison with blood endothelial tubes.

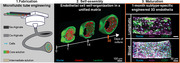

## 1 Introduction

The vascular system is composed of two complementary networks—blood and lymphatic vessels—that coordinate to regulate fluid balance, orchestrate immune surveillance, and sustain tissue homeostasis [1]. These networks share a common anatomical blueprint. Both are built from highly polarized endothelial monolayers that encase a central lumen and form hierarchically branched architectures ranging from micrometer‐scale capillaries to millimeter‐scale conduits [1]. Together, they create a contiguous three‐dimensional endothelial interface distributed throughout the body [2, 3].

Lymphatic endothelial cells (LECs) arise from a dedicated developmental lineage and are regulated by distinct transcriptional, metabolic, and signaling mechanisms [4, 5, 6]. Compared with blood vascular endothelial cells, LECs form vessels that are more permeable. They are uniquely equipped for macromolecular uptake and interstitial fluid drainage. This enhanced transport capacity is central to lymphatic physiology and underlies the contribution of the lymphatic vasculature to immune cell trafficking, inflammation, fibrosis, and tumor progression [6, 7, 8, 9].

The development of the lymphatic vasculature during embryogenesis has been extensively investigated in vivo, particularly in mouse and zebrafish models [10, 11]. However, recapitulating this context‐dependent behavior in vitro remains a major experimental challenge [4, 12]. Animal models are limited by interspecies differences and restricted scalability. Two‐dimensional cultures lack physiological architecture and provide limited insight into spatial organization and mechanical cues relevant to lymphatic endothelium [12]. Three‐dimensional systems have begun to address key features of lymphatic function, including permeability [13, 14, 15], junctional organization [16, 17, 18], and sprouting behavior [19, 20, 21]. Some platforms also incorporate flow or matrix barriers to mimic interstitial drainage [22, 23]. Yet, many of these models are short‐lived and often restricted to less than 1 week of culture. This short time window limits the ability to study how the lymphatic endothelium organizes and stabilizes within a tissue‐like environment. In addition, several engineered systems rely on lymphatic‐like cells derived from pluripotent stem cells, which may lack the maturity and stability of primary adult LECs [24, 25, 26].

A central challenge is therefore to engineer a human lymphatic endothelium that is lumenized, size‐controlled, and stable over weeks, while preserving lineage‐specific identity and barrier function. An appropriate platform should offer precise geometric control and be compatible with primary human cells. It should also rely on an extracellular matrix that cells can remodel into a perivascular niche, yet that remains stable over extended culture. Ideally, the same system should also accommodate blood endothelial cells in an identical three‐dimensional context. This would enable direct comparison of lymphatic and blood endothelial lineages and help dissect how endothelial identity shapes vessel organization, matrix assembly, and transport properties.

Here, we address this challenge by combining coaxial microfluidic extrusion [27, 28, 29, 30] with a defined composite hydrogel matrix to generate three‐dimensional tubes lined by primary human dermal lymphatic endothelial cells (HDLECs). This strategy yields scalable, size‐controlled vessels with tightly defined microenvironments. Under optimized matrix conditions, HDLECs proliferate, self‐organize into lumenized monolayers, and maintain lymphatic marker expression as well as selective permeability for at least 30 days. Using immunofluorescence, we show that HDLEC tubes adopt tissue‐like architectures. Bulk RNA sequencing indicates enrichment of transcriptional programs associated with lymphatic morphogenesis, extracellular matrix organization, and maturation. The same matrix also supports long‐lived tubes formed by blood endothelial cells. This configuration enables direct, side‐by‐side comparison of lymphatic and blood endothelial lineages under identical three‐dimensional conditions. Altogether, this platform provides a tunable system to study human endothelial biology and to probe lineage‐specific structure–function relationships in defined, physiologically relevant microenvironments.

## 2 Results

### 2.1 Size‐Controlled Hydrogel Tubes Produced by Coaxial Extrusion Support Lymphatic Endothelial Self‐Organization

**FIGURE 1:**
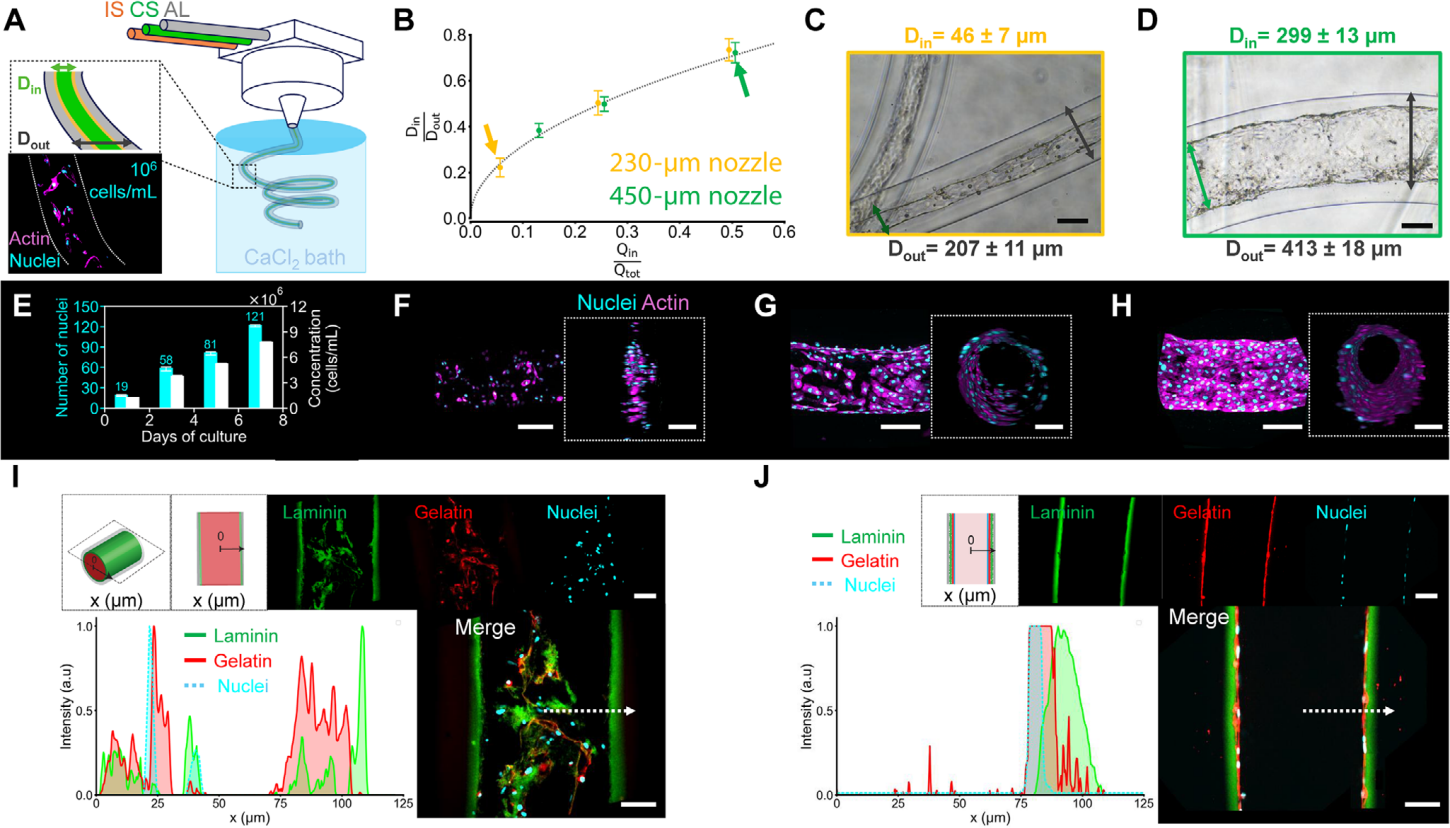
Size‐controlled hydrogel tubes supporting 3D lymphatic endothelial self‐organization. (A) Schematic of the three‐stream coaxial extrusion set‐up. The core stream (CS) contains HDLECs suspended at 10^6^ cells/mL in a composite matrix of gelatin (G, 2% w/v), Matrigel (MG, 30% v/v), hyaluronic acid (HA, 0.2% w/v), and fibrinogen (FG, 0.2% w/v). The CS is co‐extruded with a sorbitol‐based intermediate stream (IS) and an alginate shell stream (AL) into a CaCl_2_ bath, where the alginate crosslinks to form a stable tubular construct. Inset, confocal cross‐section showing a resulting tube with HDLECs lining the inner wall (nuclei, cyan; actin, magenta). (B) Flow‐controlled lumen scaling. The diameter ratio D_in_/D_out_ is plotted as a function of the inner‐to‐total flow ratio Q_in_/Q_tot_ for two nozzle sizes (230 µm, orange; 450 µm, green). Data are presented as mean ± SD (n ≥ 3 independently extruded tubes per condition). The dashed curve shows the mass‐conservation prediction $D_{in}/\;D_{out}=\sqrt{\left(Q_{in}/Q_{tot}\right)}$ (Equation 1**),** with Q_in_ = Q_CS_ + Q_IS_ and Q_tot_ = Q_CS_ + Q_IS_ + Q_AL_. Orange and green arrows highlight the specific conditions corresponding to the representative tubes shown in (C,D), respectively. (C,D) Brightfield images of lymphatic tubes at day 7 extruded under the conditions indicated by the arrows in (B). (C) 230 µm nozzle, yielding D_in_ = 46 ± 7 µm and D_out_ = 207 ± 11 µm (orange frame). (D) 450 µm nozzle, yielding D_in_ = 299 ± 13 µm and D_out_ = 413 ± 18 µm (green frame). D_in_ and D_out_ were quantified at three positions per tube (≥ 1 mm apart) across at least three independently extruded tubes per condition. (E) Quantification of HDLEC proliferation within 200 µm‐diameter tubes. Bars show the number of nuclei per 500 µm‐long segment (left axis) and the corresponding cell concentration (right axis) from day 0 to day 7 (mean ± SD, n ≥ 3 tubes per time point). (F–H) Confocal maximum intensity projections of longitudinal and cross‐sectional views at successive time points (actin, magenta; nuclei, cyan). At day 1, HDLECs are dispersed throughout the core (F). At day 5, cells elongate and align tangentially to the lumen, with partial coverage of the inner wall (G). By day 7, HDLECs form a continuous circumferential monolayer along the alginate interface, and the core region is largely devoid of cells (H). (I,J) Equatorial confocal sections of the composite matrix at day 1 (I) and day 7 (J), stained for laminin (green), gelatin (red), and nuclei (cyan), with the imaging plane schematized above each panel. At day 1, laminin and gelatin are broadly distributed within the core. By day 7, laminin is concentrated near the alginate wall, gelatin occupies an intermediate zone, and nuclei accumulate in an inner ring adjacent to the lumen. One‐dimensional fluorescence intensity profiles were extracted along the white dotted line shown in the merged images, oriented from the lumen toward the alginate shell (lumen‐to‐wall direction). Profiles were obtained from representative equatorial confocal sections and confirmed across at least three independently extruded tubes per condition. Scale bars: 100 µm (C–H), 50 µm (I–J and insets).

We used a coaxial extrusion method, inspired by [27] and adapted from the capsule‐forming hydrogel technique described in [31], to create 3D lymphatic endothelium in vitro. Three concentric streams are focused into a single jet that is continuously extruded into a CaCl_2_ bath, where the alginate shell crosslinks instantaneously. The triple‐coaxial nozzle delivers three concentrically arranged streams: the innermost core stream (CS) containing the cell‐matrix mixture, a middle intermediate stream (IS) of sorbitol solution that separates the core from the outer phase, and the outermost alginate stream (AL). A schematic cross‐sectional representation of this arrangement is provided in Figure S1. This process yields centimeter‐scale hydrogel tubes in which a porous alginate shell encloses a cell‐laden core (Figure 1A). The approach, previously applied for cell confinement in mechanobiology [27, 28, 29, 30], provides tight control over tube geometry.

Primary human dermal lymphatic endothelial cells (HDLECs) were suspended at 10^6^ cells/mL in a defined extracellular matrix (ECM) composed of gelatin (G, 2% w/v), Matrigel (MG, 30% v/v), hyaluronic acid (HA, 0.2% w/v), and fibrinogen (FG, 0.2% w/v). This G/MG/HA/FG mixture served as the core phase and was co‐extruded with a sorbitol‐based intermediate solution and an outer sodium alginate solution through a three‐capillary coaxial nozzle (CS/IS/AL).

We first examined how extrusion parameters set the lumen size. The internal diameter was tuned by adjusting the relative flow rates of the core (CS), intermediate (IS), and alginate (AL) streams at constant total flow (4 mL/h). Increasing the ratio of inner to total flow (Q_in_/Q_tot_) leads to an increase of the ratio D_in_/D_out_ for two different nozzle sizes (230 and 450 µm). This scaling law can be simply explained by mass‐conservation (Equation 1, provided in the caption of Figure 1B). Brightfield images illustrate a small‐lumen tube obtained with the 230 µm nozzle and a large‐lumen tube obtained with the 450 µm nozzle (Figure 1C,D; Figures S2 and S3). Across all conditions, inner diameters ranged from ∼50 to ∼300 µm, consistent with human lymphatic vessels in vivo [32, 33, 34].

We then examined how HDLECs proliferate and self‐organize within these geometrically defined tubes. Over a 7 day culture period, nuclei counts revealed a robust increase in cell number (Figure 1E): cell density rose from ∼10^6^ cells/mL at day 0 to 7 × 10^6^ cells/mL by day 7. The corresponding apparent doubling time of ∼30–40 h is consistent with reported proliferation rates for primary lymphatic endothelial cells [35, 36, 37]. Confocal cross‐sections acquired at matched positions along the tubes showed a reproducible morphogenetic sequence: initially, cells remained dispersed throughout the core with only partial coverage (Figure 1F); at intermediate stages, they elongated and aligned tangentially, forming a quasi‐continuous inner layer while still occupying the bulk of the shell (Figure 1G); by day 7, HDLECs had assembled into a continuous circumferential monolayer along the alginate wall, with homogeneous nuclear distribution and cortical actin enrichment at the lumen interface (Figure 1H). Thus, a randomly seeded HDLEC suspension can robustly self‐organize into a stable, lumenized endothelial tube within 1 week under appropriate matrix conditions.

Because this morphogenesis is tightly coupled to the ECM, we next sought to define the minimal composition required to sustain it and to determine how the matrix is remodeled over time. Previous reports indicate that gelatin (G), Matrigel (MG), hyaluronic acid (HA), and fibrinogen (FG) each influence endothelial morphogenesis and matrix remodeling [30, 36, 38, 39, 40, 41, 42]. We therefore compared 1‐, 2‐, 3‐, and 4‐component formulations at fixed total polymer concentration in two configurations: alginate‐shelled tubes and unconstrained spherical domes that are used as alginate‐free 3D ECM droplet controls [43, 44, 45] (Figures S4 and S5; Tables S1 and S2). Across this series, only the four‐component G/MG/HA/FG matrix consistently supported stable vessel‐like monolayers, and it was adopted as the reference condition for all subsequent experiments.

We finally analyzed the temporal organization of this composite ECM within the tubes. At day 1, laminin and gelatin were randomly distributed throughout the core, and HDLECs showed only modest enrichment at the lumen interface (Figure 1I). By day 7, the tissue displayed a stratified architecture: nuclei formed a continuous inner ring, laminin became concentrated at the luminal boundary, and gelatin localized to an intermediate zone between the cells and the alginate wall (Figure 1J). Radial intensity profiles across the wall confirmed this segregation, consistent with active cell‐mediated remodeling. Rhodamine‐labeled gelatin progressively disappeared from the core between day 0 and day 30, leaving a faint residual lining near the alginate interface at late time points (Figure S6; Movies S1 and S2). An extended time course, including additional intermediate stages and cell‐free controls, is presented in Figures S7–S9.

Taken together, these results show that, within G/MG/HA/FG tubes, HDLECs rapidly proliferate, reorganize the composite matrix, and establish a size‐controlled, stratified, and lumenized monolayer over the 1st week of culture.

### 2.2 Engineered 3D Lymphatic Endothelium Reaches Long‐Term Homeostasis After Early Tube Formation

G/MG/HA/FG tubes synergistically support HDLEC proliferation, matrix remodeling, and self‐organization into a stratified, lumenized monolayer by day 7. We then asked how these engineered tissues behave over longer time scales. Our goal was to determine whether the 3D lymphatic endothelium evolves from a growth‐prone phase into a low‐turnover state comparable to mature lymphatic vessels in vivo [4, 46]. To address this, we monitored proliferation, viability, and tissue architecture from day 5 to day 30 in G/MG/HA/FG tubes.

**FIGURE 2:**
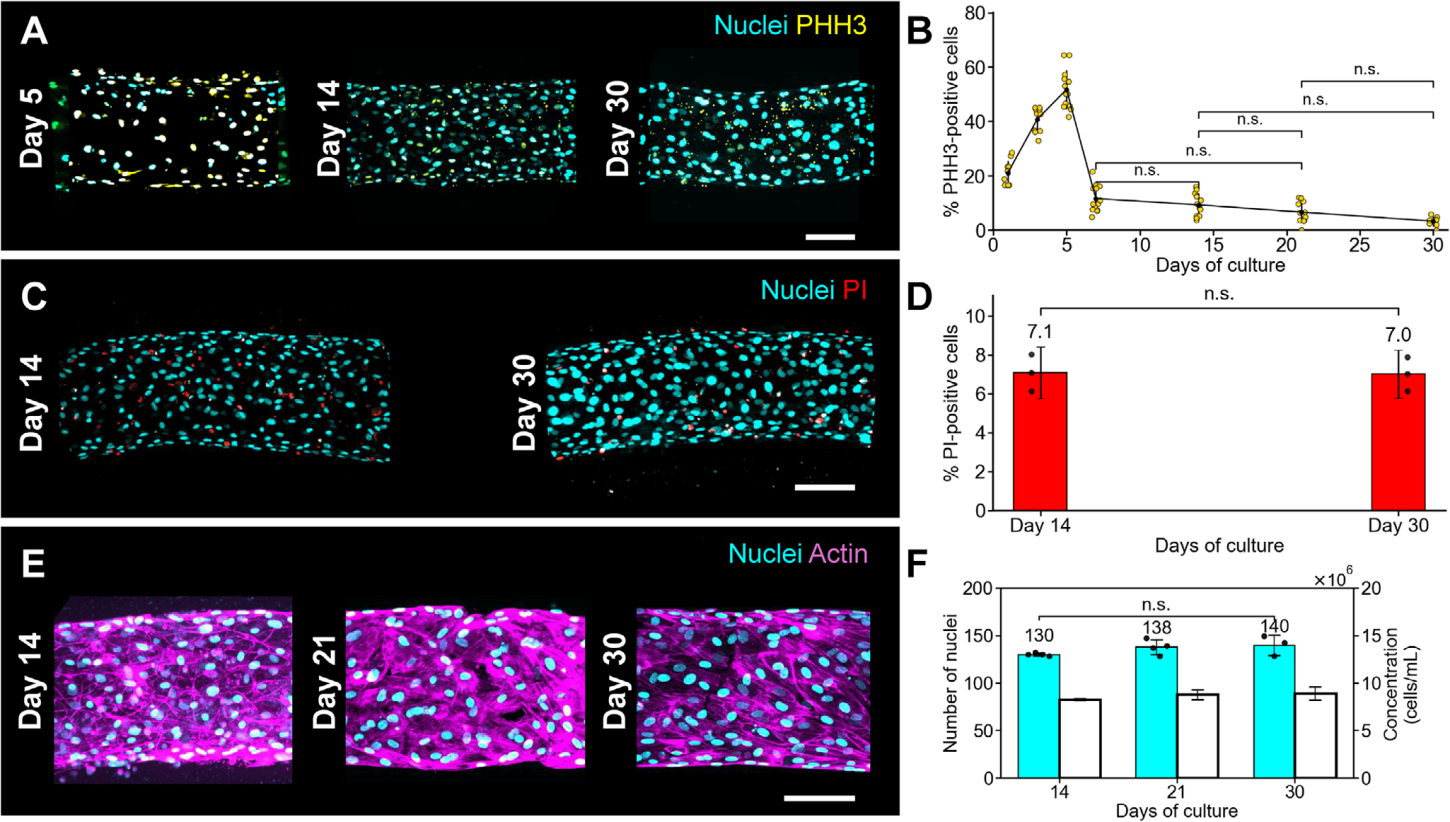
Proliferation dynamics, viability, and structural stability of 3D lymphatic endothelium over one month. (A) Maximum‐intensity projections of HDLEC tubes at days 5, 14, and 30 stained for nuclei (cyan) and phospho‐histone H3 (PHH3, yellow), illustrating the progressive decline in mitotic activity. (B) Time course of PHH3‐positive cells expressed as a percentage of total nuclei from days 1 to 30. Symbols represent mean ± SD (n ≥ 3 constructs per time point). Horizontal bars indicate pairwise comparisons; one‐way ANOVA with Tukey's post hoc test; n.s., not significant. (C) Maximum‐intensity projections at days 14 and 30 stained for nuclei (cyan) and propidium iodide (PI, red), showing rare PI‐positive cells at both time points. (D) Quantification of PI‐positive cells at days 14 and 30. Bars indicate mean ± SD (n ≥ 3 constructs per time point); numerical values above bars correspond to the mean percentage of PI‐positive cells. No significant difference was detected between days 14 and 30 (unpaired two‐tailed *t*‐test, n.s.). (E) Longitudinal confocal maximum‐intensity projections of HDLEC tubes at days 14, 21, and 30 stained for nuclei (cyan) and cortical actin (magenta), showing a continuous luminal monolayer with preserved organization over time. (F) Quantification of nuclear content in HDLEC tubes at days 14, 21, and 30. Cyan bars show the number of nuclei per 500 µm‐long segment (left axis); black bars show the corresponding cell concentration (right axis). Values are mean ± SD (n ≥ 3 constructs per time point); numbers above cyan bars indicate mean nuclei counts per segment. Neither nuclei numbers nor the cell concentrations differed significantly across time points (one‐way ANOVA, n.s.). Scale bars: 100 µm (A, C, E).

Mitotic activity, assessed by phospho‐histone H3 (PHH3) staining, was high at day 5 (52 ± 7% PHH3‐positive cells). It dropped sharply by day 14 (9 ± 4%) and was almost extinguished by day 30 (3 ± 2%) (Figure 2A,B) [47]. As expected, Ki‐67 staining followed a similar trend, with strong early expression that gradually declined toward day 30 (Figure S10). Thus, the proliferative burst associated with lumen formation is followed by a regime of minimal mitotic activity.

This decline in proliferation was not accompanied by loss of viability. Propidium iodide (PI) staining showed that the fraction of dead cells remained low and essentially unchanged between days 14 and 30 (7 ± 1% and 7 ± 1%, respectively; Figure 2C,D). The constructs, therefore did not exhibit delayed necrosis or apoptosis over this interval.

We next examined whether cell numbers and tissue architecture remain stable once proliferation has slowed. Confocal imaging of longitudinal sections stained for nuclei and cortical actin at days 14, 21, and 30 revealed a preserved cylindrical architecture and a continuous luminal lining, with HDLECs maintained as a monolayer along the tube wall (Figure 2E). Quantification of nuclei on maximum intensity projections from transverse optical sections showed that both the number of nuclei per field of interest (500 µm‐long tube segment) and the corresponding estimated cell concentrations remained stable between days 14, 21, and 30, with concentrations on the order of 8–9 × 10^6^ cells/mL throughout this interval (Figure 2F). After the initial expansion described in Figure 1, the tissue thus enters a steady‐density regime.

To characterize behavior beyond day 30, HDLEC constructs were analyzed at days 40 and 50. Confocal imaging showed sustained cortical actin organization and a uniform nuclear distribution along the tube (Figure S11A,B,D,E). Viability assays indicated that cell death remained low at these later time points (8 ± 4% and 7.5 ± 3% PI‐positive cells at days 40 and 50, respectively; Figure S11C,F). Ki‐67 staining confirmed persistently low proliferative activity (6 ± 1% and 7.5 ± 4% at days 40 and 50, respectively; Figure S11G). Together, these data are consistent with a homeostatic equilibrium observed in native lymphatic tissues, in which structural integrity and overall cell density are maintained despite ongoing molecular and cellular turnover [4, 46].

We finally asked whether this long‐term organization is specific to HDLECs. We thus tested the behavior of blood endothelial cells in the same matrix mix. HDMECs and HUVECs were encapsulated in G/MG/HA/FG tubes and cultured in parallel. Brightfield imaging showed that the cylindrical geometry was preserved from day 7 to day 30, with progressive darkening of the inner wall, indicative of increasing cell coverage (Figure S12A,D). At day 30, confocal imaging revealed that both HDMEC‐ and HUVEC‐based constructs formed continuous luminal monolayers enriched in cortical actin and regularly spaced nuclei, with complete coverage in transverse sections (Figure S12B,E). Measured cell densities reached approximately 9.6 × 10^6^ cells/mL for HDMECs and 1.4 × 10^7^ cells/mL for HUVECs, values similar to or slightly higher than those in HDLEC tubes (9 ± 0.6 × 10^6^ cells/mL at day 30, Figure 2F), yet no multilayering or architectural disruption was detected.

Overall, these findings indicate that the 3D lymphatic endothelium formed within the G/MG/HA/FG matrix reaches a homeostasis‐like state characterized by low proliferative activity, limited yet constant cell death, stable cell density, and preserved structural integrity. The latter are key hallmarks of mature lymphatic tissues in vivo [4, 48, 49]. In parallel, the same engineered tubular platform supports long‐lived monolayers of blood endothelial cells. This shared context enables direct qualitative and quantitative comparison of endothelial identity and matrix organization under identical culture conditions.

### 2.3 3D Lymphatic Endothelium Maintains Lymphatic Identity at Structural and Transcriptional Levels

Having established a long‐term, low‐turnover state in HDLEC tubes, we asked whether the engineered constructs maintain a lymphatic‐specific identity. We also examined whether endothelial cells remodel their surroundings to generate their own perivascular matrix. We focused on HDLECs and their blood microvascular counterpart, HDMECs, cultured in parallel in G/MG/HA/FG tubes.

**FIGURE 3:**
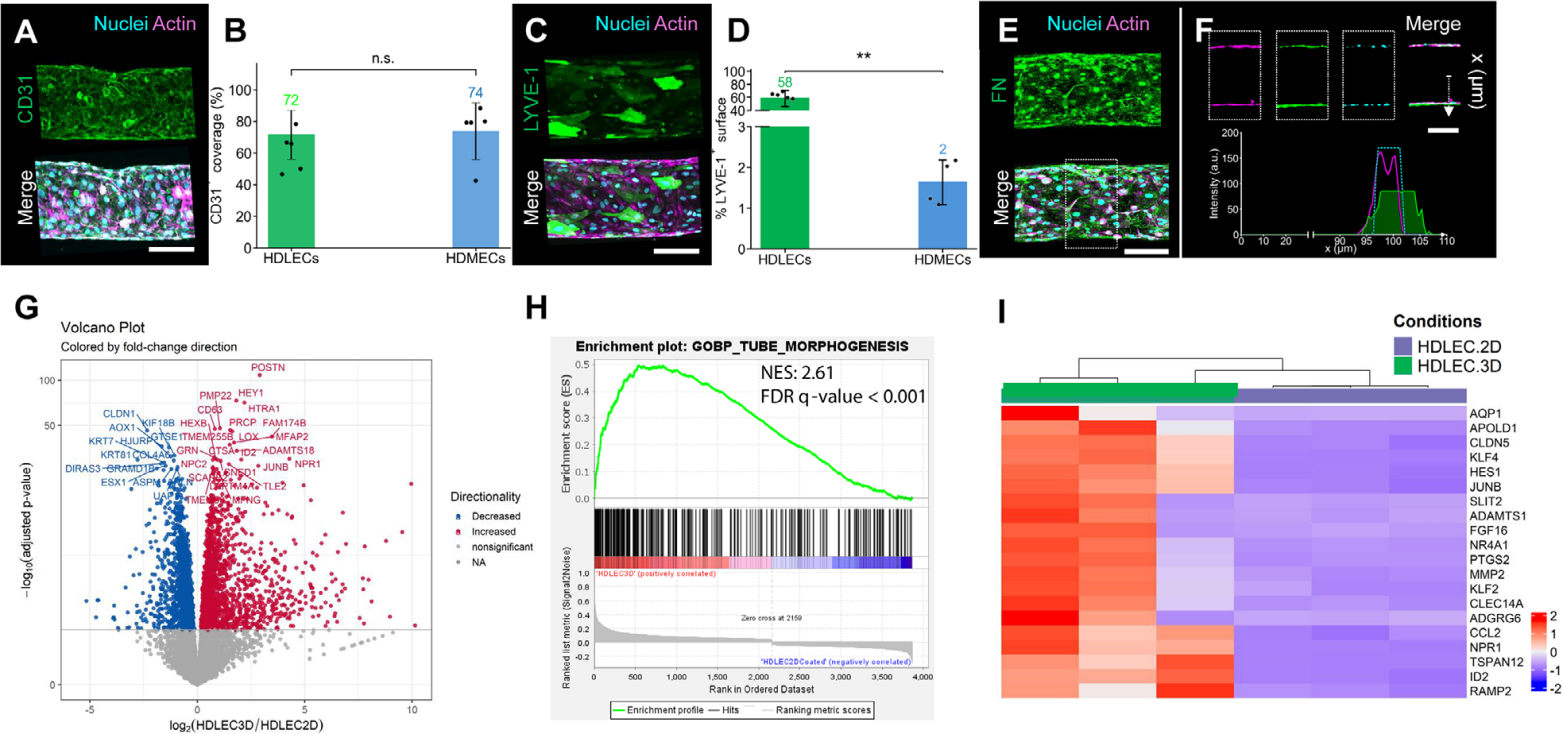
Lymphatic identity, fibronectin deposition, and transcriptomic enrichment of 3D morphogenesis genes in HDLEC tubes. (A) Confocal maximum intensity projection of an HDLEC tube at day 30 stained for CD31 (green), actin (magenta), and nuclei (cyan), showing a continuous CD31^+^ luminal monolayer. (B) Quantification of CD31^+^ coverage along the luminal contour in HDLEC and HDMEC tubes. Bars show mean ± SD, dots represent individual constructs (n ≥ 3 per condition); numbers above bars indicate mean coverage values (%). No significant difference was detected between HDLEC and HDMEC tubes (two‐tailed Mann–Whitney U test, n.s.). (C) Confocal maximum intensity projection of an HDLEC tube stained for LYVE‐1 (green), actin (magenta), and nuclei (cyan), illustrating heterogeneous LYVE‐1 expression along the monolayer. (D) Fraction of luminal surface covered by LYVE‐1^+^ regions in HDLEC and HDMEC tubes. Bars show mean ± SD, dots represent individual constructs (n ≥ 3 per condition); numbers above bars indicate mean LYVE‐1^+^ surface (%). LYVE‐1^+^ coverage is significantly higher in HDLEC than in HDMEC tubes (^**^ *p* < 0.01, two‐tailed Mann–Whitney U test). (E) Confocal single‐plane equatorial (XY) optical section of an HDLEC tube stained for fibronectin (FN, green), actin (magenta), and nuclei (cyan). FN is not part of the initial G/MG/HA/FG formulation and is therefore used as a readout of endothelial ECM deposition. The dashed box indicates the region analyzed in (F). (F) Radial localization of signals at the tube wall. Top: single‐plane equatorial (XY) views extracted from the boxed region in (E), shown as individual channels (actin, FN, nuclei) and as a merge. The arrow indicates the radial line used for profiling, sampled from the lumen toward the alginate wall (*x* = 0 at the lumen side). Bottom: corresponding one‐dimensional fluorescence intensity profiles measured along this radial line for actin, FN, and nuclei, showing FN enrichment basal to the endothelial layer, consistent with FN deposition at the cell–matrix interface. (G) Volcano plot of differentially expressed genes between HDLEC 3D and HDLEC 2D. Genes with adjusted p‐value < 0.05 are colored in red (upregulated, log2 fold‐change > 0) or blue (downregulated, log2 fold‐change < 0), regardless of fold‐change magnitude, to represent all statistically significant transcriptional differences. Non‐significant genes are shown in gray. Gene labels are displayed for transcripts with −log10(adjusted p‐value) > 25. (H) GSEA enrichment plot for tube morphogenesis signature. Normalized Enrichment Score (NES) and False Discovery Rate (FDR) q‐value are indicated on the plot. The enrichment plot displays the running enrichment score across the ranked gene list, with the peak position reflecting the point of maximal enrichment. A positive NES indicates that members of the gene set are enriched among genes upregulated in HDLEC 3D relative to HDLEC 2D. The barcode below the curve indicates the position of each gene set member in the ranked list. The leading‐edge subset, corresponding to genes to the left of the peak, represents the core set of genes driving the enrichment signal and includes key regulators of tube morphogenesis and vascular organization. (I) Heatmap of the top 20 leading‐edge genes from the tube morphogenesis signature presented in (H), comparing HDLEC 3D (right columns) with HDLEC 2D (left columns). Each column represents an individual biological replicate. Expression values are VST‐normalized, and Z‐score scaled row‐wise; red indicates relative upregulation and blue indicates relative downregulation for each gene across conditions. Hierarchical clustering was applied to both genes and samples. Scale bars: 100 µm (A, C, E, and F).

Confocal imaging at day 30 revealed that both cell types formed dense, cohesive monolayers lining the lumen, with strong cortical actin and continuous junctional CD31 staining (Figure 3A). CD31 (PECAM‐1) staining was used as a marker of endothelial identity and intercellular adhesion rather than as an indicator of tight junction organization. Quantification of CD31‐positive luminal coverage (72 ± 15% for HDLECs; 74 ± 18% for HDMECs) confirmed the presence of a largely continuous endothelial monolayer along the lumen, with regional variability reflecting the tubular geometry and local cell density (Figure 3B). In this 3D geometry, both lymphatic and blood microvascular endothelium therefore establish similarly continuous CD31^+^ junctional networks.

While examining lymphatic‐specific markers, we found that their expression was predominantly detected in the HDLEC condition. LYVE‐1 staining was heterogeneous but clearly enriched along the HDLEC monolayer, whereas HDMEC tubes exhibited sparse and weakly positive regions (Figure 3C; Figure S13). Quantification showed that LYVE‐1–positive regions covered close to 60% of the luminal surface in HDLEC tubes but only a few percent in HDMEC tubes (Figure 3D). Additional immunostaining at day 30 showed strong podoplanin (PDPN) expression in HDLEC‐derived monolayers (Figure S14), confirming that lymphatic‐specific molecular features are maintained over time in this 3D construct.

These stable, lineage‐specific signatures prompted us to examine whether HDLECs also reconstruct a lymphatic‐like perivascular niche within the tubes. We therefore analyzed HDLEC‐derived ECM deposition at day 30. In HDLEC constructs, fibronectin (FN) appeared as a continuous band immediately beneath the endothelial monolayer (Figure 3E). To characterize this spatial organization, we extracted a single‐plane equatorial (xy) confocal section and sampled fluorescence intensities along a lumen‐to‐wall radial line within the region of interest (Figure 3E,F). The resulting profiles revealed an FN intensity peak located basal to the nuclear and actin signals, consistent with local FN deposition at the cell–matrix interface and the assembly of a perivascular‐like matrix by the cells. Together with the progressive loss of bulk gelatin described above, these observations indicate that primary human lymphatic endothelial cells do not merely persist within the original G/MG/HA/FG scaffold but actively remodel it and build a basal microenvironment that approaches an in vivo‐like perivascular niche.

To assess whether these phenotypes were reflected at the transcriptional level, we performed bulk RNA sequencing, comparing HDLECs cultured in 3D tubes (HDLEC 3D) with HDLECs maintained as standard 2D monolayers on a similarly coated matrix (HDLEC 2D). We selected day 14 to capture early 3D organization, before longer‐term maturation and cell‐deposited matrix accumulation. Gene expression profiling identified differentially expressed genes between HDLEC 3D and HDLEC 2D (adjusted p‐value < 0.05) (Figure 3G; Dataset S1). Gene set enrichment analysis revealed coordinated upregulation of tube morphogenesis‐related genes (Figure 3H,I) and regulation of vascular development‐related genes (Figure S15) in HDLEC 3D compared with HDLEC 2D, as indicated by positive normalized enrichment scores (NES > 2, FDR q‐value < 0.001). The leading‐edge subset of each enrichment plot represents the core genes driving the signal and includes key regulators of tube morphogenesis and vascular organization. Further GSEA using C5 ontology gene sets confirmed enrichment of extracellular matrix organization (NES = 2.59, FDR q‐value < 0.001) and endothelial cell differentiation (NES = 2.06, FDR q‐value = 0.006) programs in the 3D condition (Figure S16B,C). To benchmark the HDLEC 3D transcriptional profile against human lymphatic endothelial identity, GSEA was performed using a curated LEC gene signature derived from a published single‐cell RNA‐seq dataset ([50]; 101 genes selected on the basis of logFC > 2, adjusted p‐value below the numerical precision threshold, pct_nz_group > 10, and pct_nz_group/pct_nz_reference > 2). This analysis demonstrated marked enrichment in HDLEC 3D compared with HDLEC 2D (NES = 2.11, FDR q‐value < 0.001; Figure S16A), supporting a stronger and more mature lymphatic transcriptional identity in the 3D tubular context. Among the leading‐edge genes driving this enrichment, LYVE1, MMRN1, and RAMP2 are established markers of lymphatic endothelial identity with documented roles in macromolecular uptake, perivascular matrix assembly, and lymphatic morphogenesis [51, 52, 53]. TSPAN12, also enriched in HDLEC 3D, has been implicated in endothelial junction regulation and vascular morphogenesis [54]. Additional, upregulated transcripts, including ANGPT2 and MMP2, are consistent with active matrix remodeling and structural stabilization of the tubular endothelium.

Taken together, these structural, molecular, and transcriptomic data indicate that HDLECs cultured in tubular G/MG/HA/FG matrices preserve a lymphatic identity and generate a cell‐derived perivascular matrix. In 3D, they also engage transcriptional programs associated with lymphatic tube morphogenesis and tissue‐like maturation, which are much less evident in standard 2D culture.

### 2.4 3D Lymphatic and Blood Endothelial Engineered Endothelia Develop Distinct Transcriptional Programs

Morphological and molecular analyses of HDLECs and HDMECs in 3D tubes highlighted the maintenance of their respective lymphatic and blood endothelial phenotypes. We next asked whether these inherited programs were imprinted at the transcriptional level. We thus performed bulk RNA sequencing on size‐matched HDLEC and HDMEC tubes cultured in 3D for 14 days, a time point at which both lineages have established continuous monolayers while still undergoing active remodeling.

**FIGURE 4:**
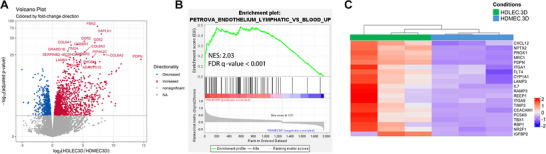
Transcriptomic profiling of 3D lymphatic and blood endothelial tubes. (A) Volcano plot of differentially expressed genes between HDLEC 3D and HDMEC 3D. Genes with adjusted *p*‐value < 0.05 are colored in red (upregulated, log2 fold‐change > 0) or blue (downregulated, log2 fold‐change < 0), regardless of fold‐change magnitude, to represent all statistically significant transcriptional differences. Non‐significant genes are shown in grey. Gene labels are displayed for transcripts with −log10(adjusted p‐value) > 20. (B) GSEA enrichment plot according to the PETROVA_ENDOTHELIUM_LYMPHATIC_VS_BLOOD_UP gene signature. NES and FDR q‐value are indicated on the plot. The enrichment plot displays the running enrichment score for this signature across the ranked gene list comparing HDLEC 3D with HDMEC 3D. The strongly positive NES and early peak position confirm systematic enrichment of lymphatic endothelial programs in HDLEC 3D. The leading‐edge subset includes canonical lymphatic markers that distinguish the HDLEC 3D transcriptional identity from that of blood endothelial cells under identical 3D conditions. (C) Heatmap of the top 20 leading‐edge genes from the PETROVA_ENDOTHELIUM_LYMPHATIC_VS_BLOOD_UP signature presented in (B), comparing HDLEC 3D (right columns) with HDMEC 3D (left columns). Each column represents an individual biological replicate. Expression values are VST‐normalized, and Z‐score scaled row‐wise; red indicates relative upregulation and blue indicates relative downregulation for each gene across conditions. Hierarchical clustering was applied to both genes and samples. Collectively, these transcriptomic analyses establish that engineered lymphatic and blood endothelial monolayers formed in the same 3D tubular scaffold adopt distinct and lineage‐related transcriptional identities. This molecular separation provides a mechanistic basis for the lineage‐specific junctional organization and barrier properties analyzed in the next section.

Gene expression profiling identified differentially expressed genes between HDLEC 3D and HDMEC 3D (adjusted p‐value < 0.05) (Figure 4A; Dataset S2). Gene set enrichment analysis revealed upregulation of lymphatic endothelium‐related genes in HDLEC 3D versus HDMEC 3D (Figure 4B,C) according to the previously established lymphatic vs. blood endothelial signature [55]. To further confirm lineage specificity in the 3D context, GSEA was performed using the curated LEC signature derived from [50]. This analysis revealed strong enrichment in HDLEC 3D versus HDMEC 3D (NES = 2.77, FDR q‐value < 0.001; Figure S17), confirming that the 3D tubular environment maintains and reinforces lymphatic endothelial identity relative to blood endothelial cells cultured under identical conditions. Among the leading‐edge genes driving this enrichment, PROX1, PDPN, FLT4, and ITGA9 are canonical markers of lymphatic endothelial identity with established roles in lymphatic lineage specification, vessel morphogenesis, and endothelial cell migration [56, 57, 58, 59]. In line with these results, similar observations were made when comparing HDLEC 3D and HUVEC 3D cultures (Figure S18 and Dataset S3).

### 2.5 3D Lymphatic and Blood Endothelial Monolayers Display Lineage‐Specific Barrier Properties

Structural, molecular, and transcriptomic analyses showed that HDLEC and HDMEC monolayers formed in 3D tubes adopt distinct identities corresponding to lymphatic and blood endothelium. In vivo, a major point of divergence between these lineages concerns barrier function, with lymphatic capillaries generally more permeable to macromolecules than blood microvessels [60, 61]. We therefore asked whether the 3D monolayers generated in our tubular scaffold reproduce such lineage‐specific differences in junctional organization and permeability.

**FIGURE 5:**
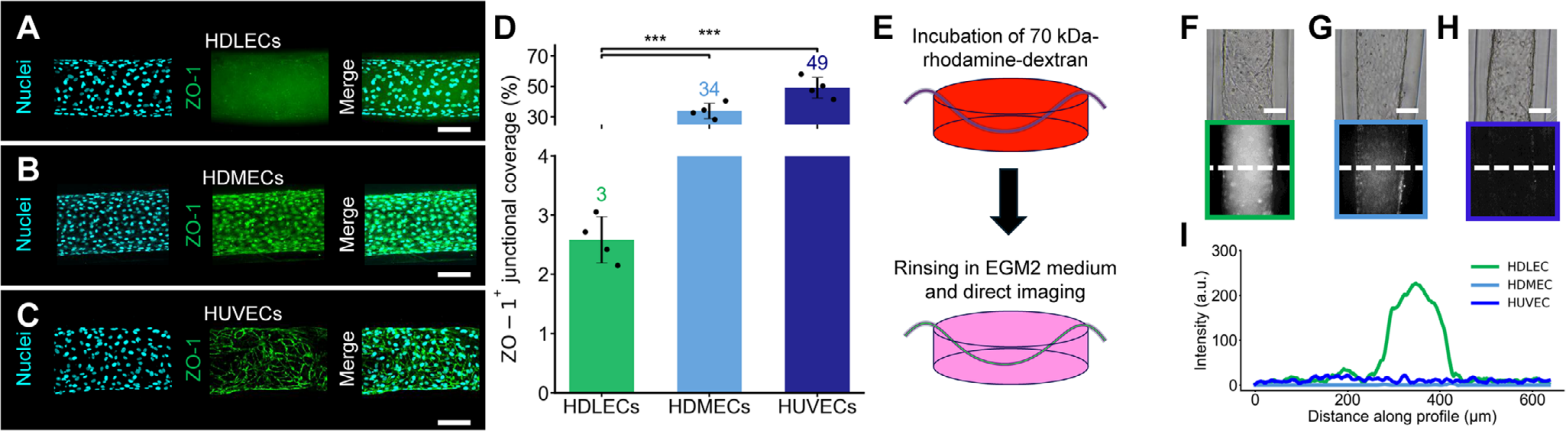
Junctional integrity and macromolecular barrier properties of 3D endothelial tubes. (A–C) Confocal maximum‐intensity projections of HDLEC (A), HDMEC (B), and HUVEC (C) tubes stained for nuclei (cyan) and the tight‐junction protein ZO‐1 (green), with merged views on the right. HDLEC tubes show sparse, discontinuous ZO‐1 labeling along the luminal surface, consistent with loose tight junction organization, whereas HDMEC and HUVEC tubes display stronger, belt‐like junctional staining. (D) Quantification of ZO‐1^+^ junctional coverage along the lumen boundary for each lineage. Bars show mean ± SD, dots represent individual constructs (n ≥ 3 per condition); numbers above bars indicate mean coverage values (%). HDLEC tubes exhibit minimal ZO‐1^+^ coverage (∼2.6%), in contrast to higher and more continuous coverage in HDMEC (∼34.0%) and HUVEC (∼49.2%) tubes (one‐way ANOVA with post hoc multiple comparisons: ^***^ *p* < 0.001 vs HDLECs; HDMEC vs HUVEC, n.s.). (E) Schematic of the macromolecular permeability assay. Tubes are incubated with 70 kDa rhodamine–dextran, rinsed in EGM2 medium, and imaged immediately to assess luminal tracer accumulation. (F–H) Brightfield (top) and corresponding fluorescence images (bottom) acquired immediately after dextran incubation for HDLEC (F), HDMEC (G), and HUVEC (H) tubes. The white dashed line marks the transect used for line‐profile analysis in (I). HDLEC tubes show a bright fluorescent band within the lumen, consistent with tracer entry, whereas HDMEC and HUVEC tubes exhibit little or no luminal signal, indicating an intact barrier to 70 kDa dextran. (I) Fluorescence intensity profiles of rhodamine–dextran along the dashed line in (F–H) (*x*‐axis: distance along the transect in µm; *y*‐axis: fluorescence intensity in arbitrary units). HDLEC tubes show a marked central peak corresponding to luminal tracer accumulation, whereas HDMEC and HUVEC profiles remain low and flat, confirming efficient exclusion of the macromolecular tracer. Scale bars: 50 µm (A–C); 100 µm (F–H).

To specifically assess tight junction organization, we stained for ZO‐1 at day 30 (Figure 5A–C) and quantified the fraction of the luminal perimeter covered by ZO‐1 signal. In contrast to the CD31‐positive monolayer continuity described above, HDLEC monolayers exhibited sparse and discontinuous ZO‐1 labeling along the luminal surface, with a mean junctional coverage of 2.6% (Figure 5D). This low ZO‐1 coverage is consistent with the permeability data showing efficient luminal entry of 70 kDa dextran, and reflects the characteristically leaky junction organization of lymphatic capillary endothelium in vivo. In contrast, HDMEC and HUVEC tubes displayed much more extensive ZO‐1 networks, with mean coverages of 34%–49%, respectively. Thus, within an identical 3D scaffold, lymphatic endothelium assembles a comparatively loose tight‐junction pattern, whereas blood endothelial monolayers form denser ZO‐1 belts. Notably, all three cell types expressed ZO‐1 in 2D monolayers (Figure S19), indicating that the differences observed in 3D arise from spatial reorganization within the tubular environment rather than from loss of expression.

We then examined whether these structural differences translate into distinct macromolecular permeability. Tubes were exposed to a 70 kDa rhodamine–dextran solution that bathed the exterior of the construct, while the tube ends remained outside the solution (Figure 5E). Under these conditions, dextran must first diffuse through the outer alginate wall and then cross the endothelial layer to enter the lumen. After washing in tracer‐free medium, we directly imaged longitudinal sections and extracted fluorescence profiles orthogonal to the tube axis (Figure 5F–I). Only HDLEC‐derived endothelium showed a clear intraluminal dextran peak, indicating substantial passage of the 70 kDa tracer across the barrier. HDMEC and HUVEC tubes, by contrast, displayed flat luminal profiles close to background, consistent with a more restrictive barrier (Figure 5I).

To assess size selectivity, we repeated the assay with 3 and 500 kDa dextrans (Figure S20) and performed parallel measurements on acellular G/MG/HA/FG tubes to determine whether the hydrogel scaffold itself imposes a diffusion barrier (Figure S21). In acellular tubes, 3 and 70 kDa dextrans freely equilibrated between the external medium and the tube lumen, whereas the 500 kDa dextran showed minimal luminal penetration, consistent with the known size‐exclusion properties of the alginate‐based matrix [62, 63]. These results confirm that the scaffold does not restrict diffusion of small and intermediate tracers and that permeability differences observed in endothelialized constructs are attributable to the endothelial monolayer. Within the molecular‐weight range for which the scaffold is permissive (3–70 kDa), only HDLEC monolayers allowed efficient luminal accumulation of the 70 kDa dextran, a size comparable to albumin. This behavior mirrors the high macromolecular permeability characteristic of lymphatic capillaries [60, 61], whereas HDMEC‐ and HUVEC‐derived tubes behave more like a tight blood vascular barrier.

Taken together, these experiments show that lymphatic and blood endothelial monolayers maintained within the same 3D tubular culture system remain long‐lived, viable, and lineage‐specific in their barrier behavior. HDLEC tubes display sparse ZO‐1 junctional coverage and selective permeability to macromolecules, whereas HDMEC and HUVEC tubes form denser ZO‐1 networks and effectively confine 70 kDa solutes. These functional differences align with the structural and transcriptional features described above and further support the view that this platform preserves key hallmarks of lymphatic and blood endothelial identity in 3D.

## 3 Discussion

This study reports a robust, tunable, and scalable platform for engineering 3D lymphatic endothelium from primary human cells. We used a microfluidic extrusion‐based technique combined with an optimized protein matrix. In this setting, HDLECs self‐organize into tubular, size‐controlled, lumenized monolayers that remain stable over time. Compared with immortalized or animal‐derived systems, this approach preserves human cellular identity and uses only small numbers of primary cells. It also offers direct control over vessel geometry. The constructs recapitulate key hallmarks of lymphatic vessels, including monolayer architecture, selective marker expression, and macromolecular permeability, while providing precise size control and long‐term maintenance.

HDLECs seeded within the tubular constructs self‐organized around a cylindrical lumen within 7 days. This process echoes early lymphatic assembly in vivo, where dispersed endothelial progenitors align, establish junctions, and form lumenized structures [64, 65]. Although culture is performed under static conditions, the in vitro monolayers remain structurally and cellularly stable for at least 30 days, surpassing the typical lifespan of many 3D endothelial models, which often degrade or delaminate within less than 2 weeks [60, 66, 67, 68]. Perfusion is not strictly required for lymphatic vessel generation, but is often considered beneficial to prevent lumen collapse and enhance functional relevance [69]. The engineered tubes form continuous lumens that should be physically compatible with direct luminal perfusion when the extremities are left open, and their mechanical stability makes them amenable to integration into perfusion‐based systems. Although controlled flow was not implemented in the present study, the defined composite ECM environment supports endothelial self‐organization and structural stability under static culture conditions. These findings indicate that matrix‐derived biochemical and biophysical cues contribute substantially to structural organization in the absence of external flow, without replacing the regulatory role of physiological shear forces. In future work, combining this matrix environment with controlled perfusion or mechanical stimulation may further enhance maturation and enable active drainage‐like functions.

Matrix composition was systematically explored to identify conditions that support both early organization and long‐term structural maturation. Among all tested formulations, only the four‐component G/MG/HA/FG mixture consistently enabled initial monolayer formation and its sustained refinement. Each component contributed distinct biophysical or biochemical cues: fibrinogen enhanced adhesion and early network formation [41]; hyaluronic acid appeared to support the maintenance of nucleated architectures, potentially synergizing with fibrinogen [36, 40]; Matrigel supplied basement membrane proteins such as laminin and collagen IV [30, 39, 42]; and gelatin promoted long‐term cohesion and facilitated ECM remodeling [38]. This four‐component matrix represents a substantial refinement over strategies in which Matrigel alone was used for vascular constructs [30]. Progressive fibronectin deposition and the emergence of a layered wall architecture indicate that HDLECs actively remodel their surroundings, mirroring matrix assembly and regulation during lymphatic development [70, 71].

Transcriptomic analyses provide a complementary view of how the 3D tubular context affects endothelial identity. In this study, we performed transcriptomic profiling at day 14 to read out early 3D, lineage‐associated programs before longer‐term maturation and cell‐deposited ECM accumulation. Compared with 2D monolayers, HDLEC tubes show strong enrichment of gene sets related to tube morphogenesis and regulation of vasculature development. Thus, the tubular G/MG/HA/FG environment does not merely sustain HDLECs but shifts them toward a more morphogenesis‐ and tissue‐organization–oriented state. In the same 3D context, HDLEC tubes are enriched for lymphatic programs, whereas HDMEC tubes preferentially express blood vascular programs. These data emphasize that lineage‐intrinsic identities remain dominant, and that 3D culture reveals differences that are partially masked in conventional 2D conditions.

Beyond structural and transcriptomic features, the model preserves hallmark transport properties of lymphatic endothelium. HDLECs sustain expression of LYVE‐1 and podoplanin. They form discontinuous junctions with sparse ZO‐1 labeling along the lumen, consistent with a role in macromolecular uptake. Consistently, HDLEC‐lined tubes permit luminal entry of 70 kDa dextran within a molecular‐weight range in which the alginate‐based shell is permissive, while still excluding a 500 kDa tracer. In contrast, blood endothelial cell–lined constructs (HDMECs and HUVECs) exhibit more continuous ZO‐1 networks, exclude the 70 kDa tracer, and lack lymphatic markers, underscoring lineage‐specific differences under identical culture conditions [46, 48, 62]. All three cell types express ZO‐1 in 2D monolayers, indicating that the reduced junctional coverage in HDLEC tubes arises from spatial reorganization of tight junctions in 3D rather than loss of expression.

Several morphological and functional features collectively suggest that the HDLEC constructs resemble initial lymphatic capillaries rather than collecting vessels. The engineered tubes display physiological diameters and relatively low cell densities, maintain expression of initial‐lymphatic markers, and exhibit loose intercellular junctions together with high macromolecular permeability. Lymphatic endothelium is known to adopt two main junctional modes: “button‐like” junctions, characterized by discontinuous junctional segments in initial lymphatics, and more continuous “zipper‐like” junctions in collecting vessels [72]. HDLECs derived from skin are expected to favor button‐like features and higher permeability. The absence of mural cells, valves, or contractile elements further supports the view that these constructs are closer to an initial lymphatic phenotype. A logical next step will be to develop complementary models of collecting lymphatics incorporating additional cell types, mechanical cues, and valve‐forming capacity.

The extrusion‐based approach itself is versatile and compatible with endothelial cells from different origins. Lymphatic and blood endothelial cells (HDLECs, HDMECs, and HUVECs) all form stable 3D monolayers with appropriate spatial organization, yet differ in cell density, junctional continuity, and permeability. These differences reflect intrinsic lineage traits rather than matrix‐related constraints, since the microenvironment is held constant. This versatility suggests that the platform can be extended to patient‐derived endothelial cells, disease‐specific mutations, or engineered lines, and combined with additional tracers or active transport readouts to probe more complex transport phenomena. The tubular geometry also enables co‐culture configurations directly relevant to immune–lymphatic interactions. Two approaches are compatible with the current platform. First, immune cells can be introduced through the open lumen after tube formation, enabling the study of direct endothelial–immune cell interactions, including adhesion and transmigration across the lymphatic monolayer under defined conditions. Second, immune cells can be incorporated within the ECM core during extrusion itself, allowing investigation of how immune cell proximity influences lymphatic morphogenesis and endothelial self‐organization from the outset. Both configurations are technically feasible, as the extrusion procedure is performed under conditions compatible with immune cell viability. These co‐culture strategies will be explored in future work.

In summary, integrating extrusion technology with a finely tuned hydrogel matrix yields an engineered 3D lymphatic endothelium that is size‐controlled, long‐lived, and built from primary human cells, while also supporting lineage‐matched blood endothelial monolayers. The system preserves structural, molecular, transcriptomic, and barrier differences between lymphatic and blood endothelium under identical 3D conditions. Beyond modeling lymphatic vessels, this platform offers a flexible and modular tool for comparative vascular studies, drug and macromolecule transport research, and investigations into endothelial adaptability and disease‐associated remodeling. It also provides a foundation on which more complex, flow‐coupled, or multicellular lymphatic models can be built.

## 4 Materials and Methods

### 4.1 Cell Culture

Primary human dermal lymphatic endothelial cells (HDLECs; PromoCell, C‐12216) and human dermal microvascular endothelial cells (HDMECs; PromoCell, C‐12212) were cultured in Endothelial Cell Growth Medium MV2 (EGM2‐MV; PromoCell, C‐22022). Human umbilical vein endothelial cells (HUVECs; Lonza, CC‐2519) were cultured in Endothelial Cell Growth Medium 2 (EGM2; PromoCell, C‐22011).

All media were supplemented with 1% penicillin–streptomycin (Gibco, 15140122). Cells were maintained in tissue culture‐treated flasks at 37°C in a humidified 5% CO_2_ incubator and used between passages 2 and 6. Before encapsulation, cells were detached with 0.05% trypsin–EDTA (Gibco, 25300054), centrifuged at 400 × g for 5 min, and resuspended in hydrogel matrices at 10^6^ cells/mL unless otherwise specified.

### 4.2 Hydrogel Formulation

Hydrogel matrices were prepared from gelatin (G, 2% w/v; porcine, Type A, Advanced BioMatrix, 5005), growth factor‐reduced Matrigel (MG, 30% v/v; Corning, 356231), low molecular weight hyaluronic acid (HA, 100–150 kDa, 0.2% w/v; Advanced BioMatrix, 5003), and human fibrinogen (FG, 0.2% w/v; Sigma–Aldrich, F8630). All components except gelatin were kept at 4°C; gelatin was added immediately before encapsulation to avoid premature gelation.

Twelve matrix formulations were generated by combining one to four macromolecular components: G, MG, HA, and FG, at respective fixed final concentrations 2% w/v, 30% v/v, 0.2% w/v, 0.2% w/v when present. Acellular matrices were prepared in parallel for control experiments.

For experiments tracking matrix remodeling by gelatin (Figure 1I,J; Figure S6; Movies S1 and S2), a fraction of the gelatin was pre‐labeled with rhodamine according to the manufacturer's instructions, while keeping the total gelatin concentration at 2% w/v.

### 4.3 Coaxial Extrusion of Tubular Constructs

The coaxial extrusion setup was adapted from previous encapsulated hydrogel systems [27, 29, 30]. A custom triple coaxial head was 3D‐printed (EnvisionTEC D4K Pro, HTM 140 V2 resin). Three syringe pumps (neMESYS low‐pressure module, CETONI) independently controlled the core, intermediate, and shell flows.

The core stream (CS) contained the cell–hydrogel mixture (G/MG/HA/FG or variants) at 4°C–7°C, with cells at 10^6^ cells/mL. The intermediate stream (IS) was 0.6 m sorbitol (Sigma–Aldrich, S1876), and the alginate shell stream (AL) was 2% sodium alginate (Alliance Gums & Industries, I1G80‐E401), sterilized by autoclaving.

Extrusion was performed into a 100 mm CaCl_2_ bath at 37°C to rapidly crosslink the alginate. Unless otherwise stated, flow rates were 2.0 mL/h (shell), 1.0 mL/h (intermediate), and 1.0 mL/h (core). Two outer nozzle diameters, 450 and 230 µm, were used to span a broad range of lumen sizes. Within 10 min of extrusion, tubes were transferred to 60 mm Petri dishes (Corning, CLS430166) containing 5 mL of prewarmed endothelial medium and placed at 37°C, 5% CO_2_.

By systematically varying the ratio Q_in_/Q_tot_, we found that the inner diameter D_in_ for both 230 and 450 µm nozzles closely followed a simple mass‐conservation model (Equation 1; Figure 1B).

### 4.4 Culture Conditions and Experimental Time Course

After extrusion, tubes were cultured in 60 mm Petri dishes with 5 mL of endothelial medium per dish. HDLEC and HDMEC constructs were maintained in EGM2‐MV (PromoCell, C‐22022); HUVEC constructs were maintained in EGM2 (PromoCell, C‐22011). Media were renewed every 48 h by gentle aspiration and replacement. No perfusion or mechanical agitation was applied.

Constructs were analyzed at days 0, 1, 2, 3, 5, 7, 14, 21, and 30. In selected experiments, additional endpoints at days 40 and 50 were included. At each time point, 1 cm segments from at least three independently extruded tubes per condition were collected for viability assays, permeability measurements, quantitative imaging, or fixation and immunostaining.

### 4.5 Cell Viability Assays

Cell viability was assessed at days 7, 14, 21, 30, 40, and 50 using the LIVE/DEAD Viability/Cytotoxicity Kit (Thermo Fisher Scientific, L3224). Tube segments (1 cm) were transferred into PLL‐coated 60 mm dishes (poly‐L‐lysine; Gibco, A3890401) to minimize movement, then incubated for 30 min at 37°C in phenol red–free DMEM containing 2 µm calcein‐AM and 4 µm ethidium homodimer‐1. After a gentle rinse in fresh medium, constructs were imaged immediately on the spinning‐disk confocal system (see Brightfield and confocal imaging).

For the quantitative viability measurements reported in Figure 2C,D, propidium iodide (PI) was used. Tube segments were incubated for 15 min at 37°C in culture medium containing 1 µg/mL PI, rinsed three times in fresh medium, and imaged under identical settings. PI^+^ nuclei were quantified as a percentage of total Hoechst^+^ nuclei.

### 4.6 Immunofluorescence Staining

At defined time points, 1 cm tube segments were transferred to 1.5 mL microtubes and fixed in 4% paraformaldehyde (PFA; Fisher Scientific, F/1501/PB17) in DMEM (PAN‐Biotech, P04‐05545) for 1 h at room‐temperature under gentle agitation. Samples were washed three times with DMEM and stored at 4°C until staining.

For staining, samples were permeabilized for 30 min in DMEM with 1% Triton X‐100 (Sigma–Aldrich, T8787), then blocked for 1 h in DMEM containing 1% BSA (Sigma–Aldrich, A9647) and 10% FBS (Clinisciences, FBS Xtra, FBS‐16A). All incubations were performed under gentle orbital shaking (40 rpm) to facilitate diffusion into the lumen.

Primary antibodies were diluted in blocking buffer supplemented with 0.1% Triton X‐100 and 2% FBS and incubated overnight at 4°C. The following primary antibodies were used:

– CD31 (polyclonal sheep IgG, 1:200; BioTechne, AF806)
– LYVE‐1 (rabbit monoclonal, 1:500; Abcam, ab219556)
– Podoplanin / PDPN (mouse monoclonal, 1:500; MBL Life Science, 8F11)
– Fibronectin (rabbit polyclonal, 1:500; Abcam, ab2413)
– ZO‐1 (mouse monoclonal, 1:500; Thermo Fisher Scientific, ZO1‐1A12)
– Phospho‐histone H3 (rabbit polyclonal, 1:500; Sigma‐Aldrich, SAB4502231)
– Ki‐67 (rabbit polyclonal, 1:500; Abcam, ab15580)
– Laminin (rabbit polyclonal, 1:200; Abcam, ab11575)

After washes in DMEM, samples were incubated overnight at 4°C with Alexa Fluor 488, 568, or 647–conjugated secondary antibodies (1:500; Thermo Fisher Scientific). F‐actin was labeled with Phalloidin–Alexa Fluor 568 (1:200; Thermo Fisher Scientific, A12380), and nuclei were counterstained with Hoechst 33342 (1 µg/mL; Thermo Fisher Scientific, H3570).

To improve imaging depth and reduce scattering, stained samples were optically cleared by sequential immersion in 30%, 60%, and 100% FUnGI solution. FUnGI was prepared as described in [73] (50% glycerol w/v, 2.5 m fructose, 2.5 m urea, 10.6 mm Tris base, 1 mm EDTA).

Cleared constructs were mounted between glass slides (RS France, BP024) with adhesive iSpacer chambers (0.25 mm or 0.15 mm; SunJin Lab, IS216 and IS015) and coverslips (Merck‐Sigma, CLS2980223), then stored at 4°C until imaging.

### 4.7 Brightfield and Confocal Imaging

Live constructs were monitored by brightfield microscopy on a Nikon Eclipse Ti inverted microscope equipped with phase‐contrast objectives and a DS‐Fi3 camera (Nikon). Images were acquired at fixed time points (days 0, 2, 5, 7, 14, 21, and 30) with identical exposure settings using NIS‐Elements software. Long‐term phase contrast and fluorescence movies (Movies S1 and S2) were acquired with an Incubascope system positioned inside a standard incubator, as described in [74].

Fixed and stained tubes were imaged by spinning‐disk confocal microscopy on a Leica DMI8 inverted microscope (Leica Microsystems) equipped with a CSU‐W1 spinning‐disk unit (Yokogawa) and a LiveSR super‐resolution module (Gataca Systems). A 20× multi‐immersion objective (Leica HC PL APO CS2, NA 0.75, working distance 0.66 mm) and a Prime 95B sCMOS camera (Photometrics) were used. Excitation was provided by 405, 488, 561, and 642 nm laser lines. Z‐stacks were acquired with a step size of 0.55 µm. Maximum intensity projections were generated in FIJI (ImageJ), with uniform adjustments to brightness and contrast across comparable datasets. No deconvolution, denoising, or nonlinear filtering was applied.

### 4.8 Image Analysis

All image analyses were performed in FIJI (ImageJ 1.53) with the MorphoLibJ plugin and, where indicated, custom Python 3.9 scripts (Spyder, Anaconda) using NumPy, pandas, SciPy, and matplotlib.

- Nuclear segmentation and cell density

Nuclei were segmented from Hoechst‐stained maximum intensity projections using Gaussian blur, Otsu thresholding, and watershed separation. For each tube, rectangular regions of interest (ROIs; 200 µm × 500 µm in the tube frame) were defined at matched positions. Cell concentration (cells/mL) was computed by relating nuclear counts to the volume of the corresponding cylindrical segment, using the measured inner diameter and the ROI length.

- Proliferation and viability markers

For PHH3, Ki‐67, and LIVE/DEAD assays, nuclei were first detected in the Hoechst channel. Marker‐positive cells (PHH3^+^, Ki‐67^+^, calcein^+^, ethidium^+^, or PI^+^) were segmented by thresholding the corresponding fluorescence channel (Otsu or fixed threshold within a dataset) followed by watershed separation. Particle analysis in FIJI provided counts for total and marker‐positive cells. Fractions were expressed as percentages of marker‐positive nuclei over total nuclei per field. Thresholds and size filters were kept constant across experiments of a given type.

- CD31 and ZO‐1 junctional coverage

CD31 and ZO‐1 junctional coverage were quantified from maximum intensity projections using a contour‐based pipeline in MorphoLibJ, applied identically to both markers. First, the luminal boundary was delineated using actin and nuclear channels, followed by global thresholding, morphological closing, and marker‐controlled watershed to obtain a binary mask of the wall. MorphoLibJ's “Label Boundaries” function was then used to extract a one‐pixel‐wide total luminal contour.

CD31^+^ or ZO‐1^+^ contours were identified by thresholding the corresponding channel and intersecting the resulting binary mask with the luminal boundary. Otsu's method was used by default; when it yielded unsatisfactory segmentation due to atypical intensity distributions, the Moments threshold was used instead. For a given marker, the selected thresholding method was defined at the experiment level and then applied unchanged across all conditions and images within that experiment. The lengths of the total contour and the positive contour were computed as the number of boundary pixels. Junctional coverage (%) was defined as:

Coverage(%)=(Lpositive/Ltotal)×100

At least three independent constructs and multiple fields per construct were analyzed per condition.

- LYVE‐1–positive luminal surface

LYVE‐1 coverage was measured as the fraction of the luminal surface area occupied by LYVE‐1^+^ regions. On maximum intensity projections, a luminal ROI was drawn based on actin and nuclear channels to encompass the endothelial lining and lumen. LYVE‐1 images were background‐subtracted and thresholded (Otsu or fixed) to obtain a binary LYVE‐1^+^ mask. The LYVE‐1^+^ surface fraction (%) was computed as LYVE‐1^+^ area divided by total luminal ROI area, multiplied by 100.

- ECM intensity profiles

For extracellular matrix components (laminin, gelatin, and fibronectin), radial fluorescence intensity profiles were extracted from confocal images to assess their spatial distribution across the tube wall. For each tube, a single line ROI was drawn from the lumen toward the alginate shell (lumen‐to‐wall direction) in the selected region of interest. Profiles were obtained from a single‐plane equatorial XY confocal section (central z‐slice), as illustrated in Figure 1I,J, and Figure 3E,F. Fluorescence intensities along this line were sampled and exported from FIJI and subsequently analyzed in Python to generate the radial intensity profile for each channel. Nuclei were used solely as spatial landmarks; accordingly, the nuclear channel was acquired to reliably detect dim nuclei across heterogeneous labeling, while allowing saturation of the brightest pixels. Given the negligible background, this approach generated high‐contrast nuclear masks for robust localization and segmentation, avoiding high‐dynamic‐range acquisition requiring multiple exposures, thereby increasing photobleaching. These profiles were used to evaluate the relative positioning of ECM signals with respect to nuclei and cortical actin and to document the stratification of laminin, gelatin, and fibronectin within the wall.

- Dextran intensity profiles

For permeability assays, fluorescence intensity profiles were extracted in FIJI along line ROIs spanning the wall and lumen and oriented orthogonally to the tube axis. For each tube, several line profiles were averaged to obtain a representative radial profile. Custom Python scripts were used to normalize intensities, identify intraluminal peaks, and compare profiles between conditions.

### 4.9 RNA Sequencing and Analysis

For transcriptomic profiling, 3D endothelial tubes were generated using the standard coaxial extrusion workflow described above, with the reference four‐component core matrix composed of gelatin (G, 2% w/v), growth factor–reduced Matrigel (MG, 30% v/v), low–molecular weight hyaluronic acid (HA, 0.2% w/v), and human fibrinogen (FG, 0.2% w/v). The core stream contained the cell–hydrogel mixture at 10^6^ cells/mL, and tubular constructs were maintained in endothelial culture medium for 14 days. For each 3D condition (HDLEC, HDMEC, and HUVEC), three independent biological replicates were generated. At day 14, the alginate shell was enzymatically removed by adding alginate lyase to the culture medium (1:100 dilution from a 4 mg/mL stock solution). Cells were then recovered, rinsed in PBS, pelleted by centrifugation, and stored at −80°C prior to shipment for RNA‐seq processing. In parallel, 2D HDLEC control cultures were prepared by coating tissue‐culture flasks with the same G/MG/HA/FG formulation (at the concentrations stated above), seeding HDLECs, and expanding cells to ∼70% confluence (typically 4 days). Three independent 2D biological replicates were prepared. Cells were harvested by trypsinization, pelleted by centrifugation, and stored at −80°C until processing. Total RNA was extracted by Azenta Life Sciences from frozen cell pellets according to the provider's standard procedures. RNA integrity and concentration were assessed using Fragment Analyzer and Qubit fluorometric assays, respectively. cDNA libraries were sequenced on an Illumina NovaSeq instrument using a paired‐end 150‐nt strategy (Azenta Life Sciences). Reads were aligned to the reference genome with the STAR aligner (Spliced Transcripts Alignment to a Reference), and gene‐level counts were generated using the Rsubread package. Raw sequencing data (FASTQ files) will be deposited in the NCBI Sequence Read Archive (SRA), and the accession number will be provided as soon as available. Differential expression analysis was performed in R using DESeq2. Gene set enrichment analysis was conducted with GSEA using the C5 ontology gene sets and the PETROVA_ENDOTHELIUM_LYMPHATIC_VS_BLOOD_UP signature from the Molecular Signatures Database; enrichment plots were generated with the same software. To generate a curated lymphatic endothelial cell signature for GSEA benchmarking, 101 highly specific LEC genes were selected from a published single‐cell RNA‐seq dataset [50] by applying the following filtering criteria: logFC > 2, adjusted p‐value below the minimum detectable computational threshold, pct_nz_group > 10, and pct_nz_group/pct_nz_reference ratio > 2. Heatmaps were generated from VST‐normalized expression data using Z‐score scaling row‐wise across samples, where red indicates relative upregulation and blue indicates relative downregulation. Hierarchical clustering was applied to both genes and samples.

### 4.10 Permeability Assays

Barrier properties were assessed using rhodamine B–conjugated dextrans of different molecular weights: 3 kDa (Invitrogen, D3307), 70 kDa (Invitrogen, D1841), and 500 kDa (HAworks, DE‐Rhodamine‐500k). Tubes were incubated for 30 min at 37°C in culture medium containing 100 µg/mL tracer. Both ends of each tube were kept fully outside the fluorescent solution so that dextran could reach the lumen only by diffusing through the alginate shell and crossing the endothelial layer.

After incubation, constructs were rinsed three times in prewarmed medium (EGM2 or EGM2‐MV, depending on cell type). Brightfield and fluorescence images were acquired on the Nikon Eclipse Ti microscope under identical settings for all conditions. Radial fluorescence profiles across the wall and lumen were extracted in FIJI and analyzed with custom Python scripts as described above.

### 4.11 Statistical Analysis

Tube diameter reproducibility was evaluated by measuring internal (D_in_) and external (D_out_) diameters at three positions per tube (≥ 1 mm apart) on N ≥ 3 independently extruded tubes per condition.

Unless otherwise indicated, data are presented as mean ± standard deviation (SD). Statistical analyses were performed in OriginPro (OriginLab, version 8) and Python 3.9 (SciPy, statsmodels, pandas, matplotlib). Normality was assessed by the Shapiro–Wilk test. Depending on distribution and design, the following tests were used:

– Two‐group comparisons: unpaired t‐test for normally distributed data; Mann–Whitney U test when normality was not met.
– Multiple‐group comparisons: one‐way ANOVA with Tukey's post hoc test when parametric assumptions were satisfied; Kruskal–Wallis test with Dunn's correction otherwise.

For time‐course analyses (for example, Ki‐67, PHH3), one‐way ANOVA with Tukey's post hoc test was applied to compare time points. Full statistical outputs are provided in Tables S3–S5.

Significance was reported using the following scheme: ^****^ *p* < 0.0001; ^***^ *p* < 0.001; ^**^ *p* < 0.01; ^*^ *p* < 0.05; n.s., *p*≥ 0.05.

For clarity, statistical annotations were limited to comparisons highlighted in each figure and/or specified in figure legends.

All experimental procedures—including matrix preparation, extrusion parameters, culture conditions, staining protocols, imaging settings, and analysis pipelines—were applied uniformly across endothelial cell types and time points, allowing robust, lineage‐specific comparisons of structural, molecular, transcriptomic, and barrier properties. Unless otherwise specified, all statistical tests were two‐sided, and significance was assessed at α = 0.05. No formal outlier exclusion was applied.

## Author Contributions

**Elsa Mazari‐Arrighi**: Conceptualization, Formal analysis, Investigation, Methodology, Visualization, Writing – original draft, Writing – review & editing. **Adeline Boyreau**: Investigation, Methodology. **Laura Chaillot**: Investigation, Methodology. **Wilfried Souleyreau**: Investigation, Methodology. **Laetitia Andrique**: Conceptualization, Investigation, Methodology. **Pierre‐Olivier Guichet**: Investigation, Methodology, Writing. **Thomas Mathivet**: Conceptualization, Methodology, Writing, Supervision. **Andreas Bikfalvi**: Conceptualization, Formal analysis, Methodology, Project administration, Resources, Supervision, Validation, Writing – review & editing. **Pierre Nassoy**: Conceptualization, Formal analysis, Funding acquisition, Methodology, Project administration, Resources, Supervision, Validation, Writing – review & editing.

## Funding

PN acknowledges support from the French National Agency for Research (ANR‐21‐CE18‐0038; ANR‐23‐CE45‐0016) and from the Institut National du Cancer (PLBIO23‐097). AB and LA acknowledge support from the French National Agency for Research (ANR‐21‐CE19‐0029 IVEON). This study also received financial support from the French government in the framework of the University of Bordeaux's France 2030 program / GPR LIGHT.

## Conflicts of Interest

The authors declare no conflicts of interest.

## Supporting information




**Supporting File 1**: adhm71307‐sup‐0001‐SuppMat.pdf.

**Supporting File 2**: adhm71307‐sup‐0002‐MovieS1.avi.

**Supporting File 3**: adhm71307‐sup‐0003‐MovieS2.avi.

**Supporting File 4**: adhm71307‐sup‐0004‐Dataset.zip.

## Data Availability

Data supporting the findings of this study are included in the article and its supplementary information. The differential expression results are provided as Supplementary Datasets 1–3. Raw sequencing data (FASTQ files) will be deposited in the NCBI Sequence Read Archive (SRA), and the accession number will be provided as soon as available in accordance with journal policy
