# Supplementary material for: A Versatile Microfluidic Extrusion‐Based Hydrogel Platform for Self‐Organization and Long‐Term Maintenance of Engineered 3D Lymphatic Endothelium
Source: Adv Healthc Mater. 2026 Jun 3;15(25):e71307. doi: 10.1002/adhm.71307 (PMC13331603; doi:10.1002/adhm.71307)
Supplement: Supplementary file 1 — Supporting File 1: adhm71307‐sup‐0001‐SuppMat.pdf. [file ADHM-15-0-s003.pdf]

Supporting information

A versatile microfluidic extrusion-based hydrogel platform for self-organization and long-term maintenance of engineered 3D lymphatic endothelium

Supplemental Figures

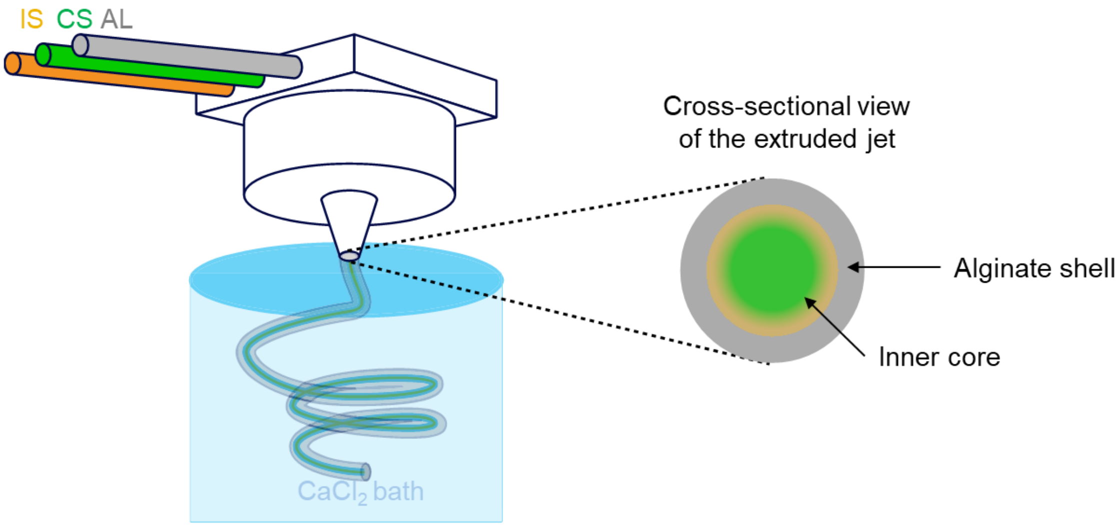

Supplementary Figure S1. Schematic representation of the triple-coaxial extrusion system used to generate hydrogel tubes

The device delivers three concentric streams through a triple-capillary coaxial nozzle: the innermost core stream (CS, cell–matrix mixture), the intermediate stream (IS, sorbitol solution), and the outermost alginate stream (AL). After extrusion into a $CaCl_2$ bath, the alginate crosslinks rapidly to form the outer hydrogel shell. The cross-sectional view illustrates the concentric arrangement of the three streams at the nozzle exit, with the CS occupying the central region, separated from the AL by the IS, which prevents premature mixing and controls the wall thickness of the resulting tube.

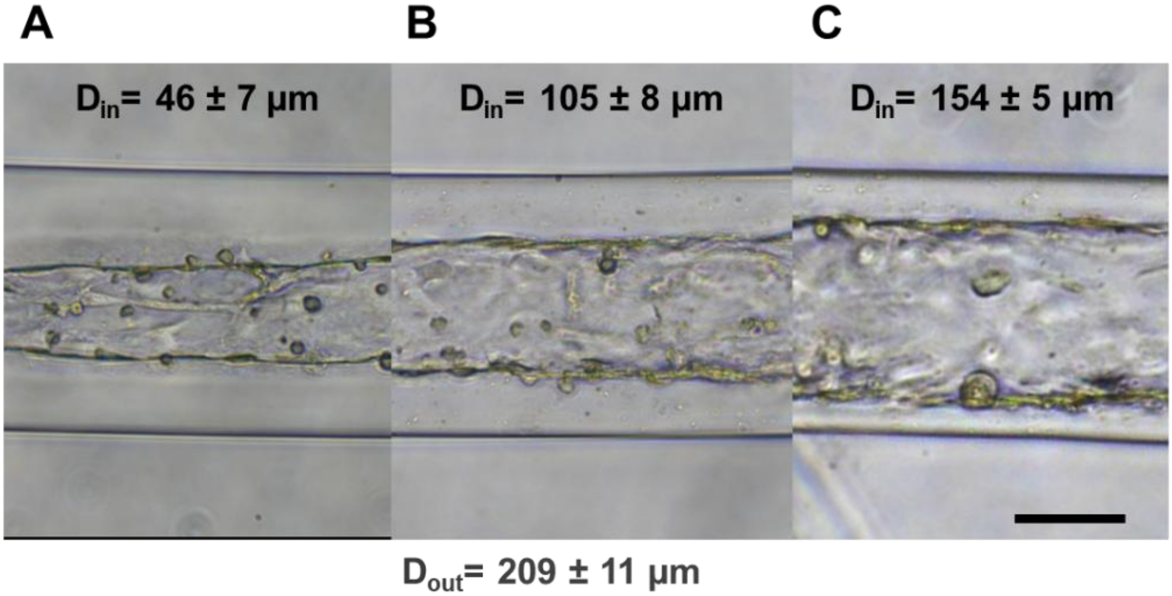

Supplementary Figure S2. Fabrication of 3D lymphatic endothelium with tunable internal diameters using a 230-µm nozzle extrusion device

(A–C) Brightfield images of constructs produced with corresponding CS/IS/AL flow rate combinations of 0.15/0.15/3.7 mL/h **(A)**, 0.5/0.5/3 mL/h **(B)**, and 1/1/2 mL/h **(C)**. According to Fig. 1, the internal diameter (D_{in}) increased with the relative contribution of the core stream, from around 50 µm to 150 µm, while the external diameter (D_{out}) remained approximately constant (209 ± 11 µm).

Scale bar: 100 µm.

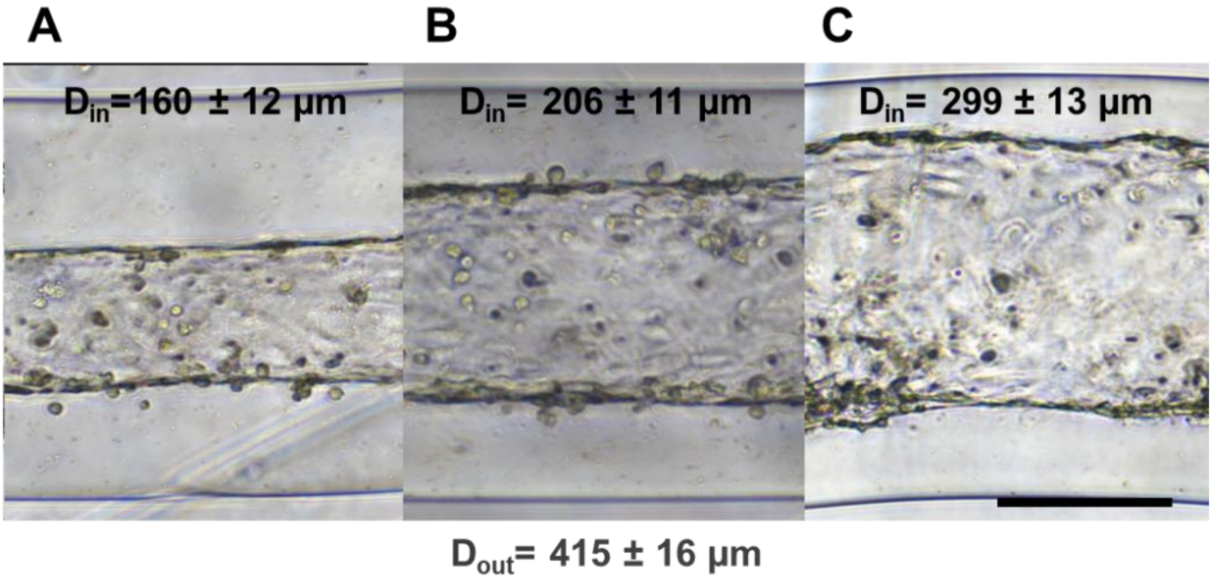

Supplementary Figure S3. Fabrication of 3D lymphatic endothelium with tunable internal diameters using a 450-µm nozzle extrusion device
(A–C) Brightfield images of constructs produced with corresponding CS/IS/AL flow rate combinations of 0.25/0.25/3.5 mL/h **(A)**, 0.5/0.5/3 mL/h **(B)**, and 1/1/2 mL/h **(C)**. According to Fig. 1, the internal diameter (D_{in}) expanded with the relative contribution of the core stream, from around 160 µm to around 300 µm, while the external diameter (D_{out}) remained stable (415 ± 16 µm).
Scale bar: 200 µm.

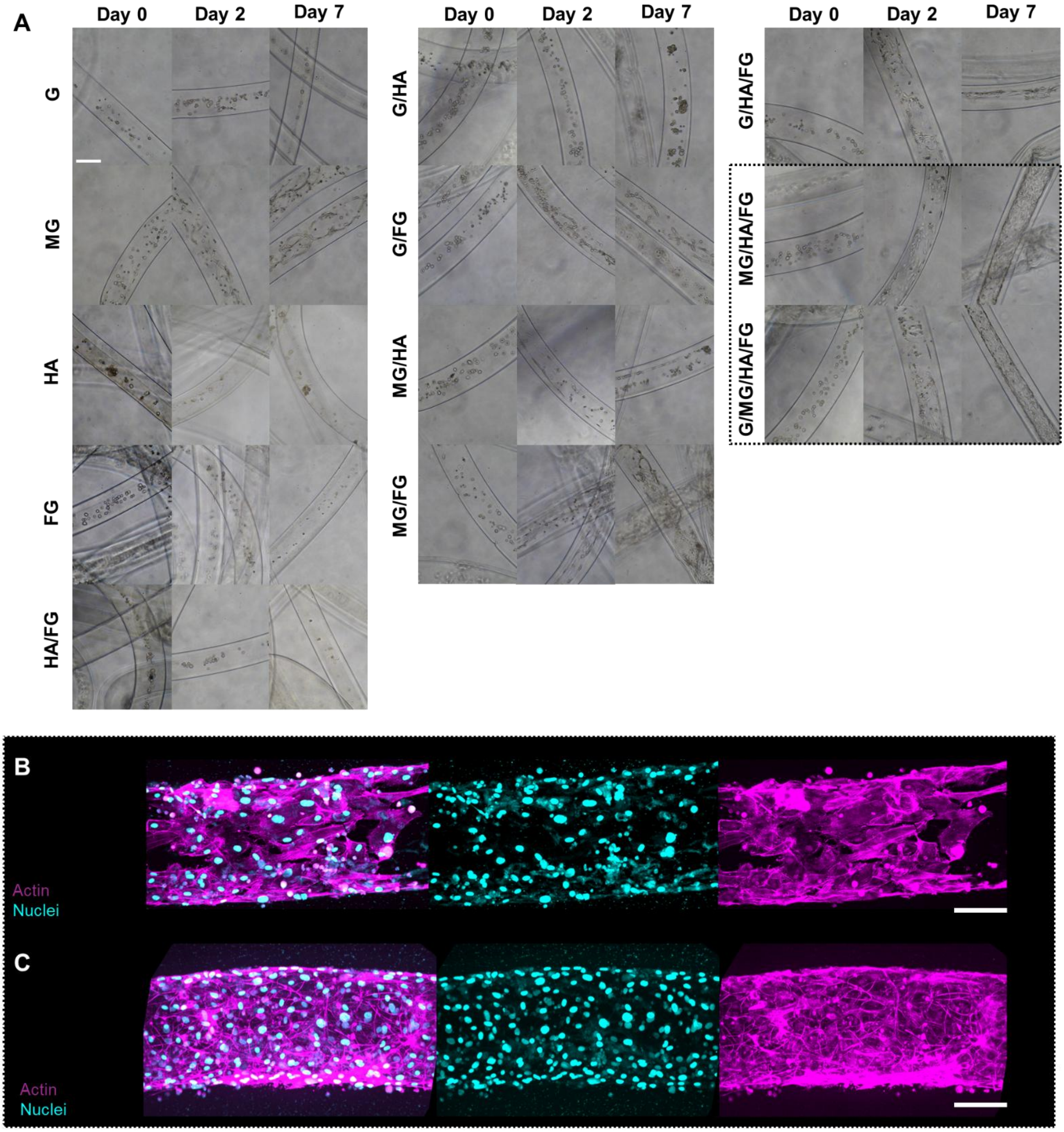
A
Day 0
Day 2
Day 7
G
MG
HA
FG
HA/FG
G/HA
G/FG
MG/HA
MG/FG
G/HA/FG
MG/HA/FG
G/MG/HA/FG
B
Actin
Nuclei
C
Actin
Nuclei

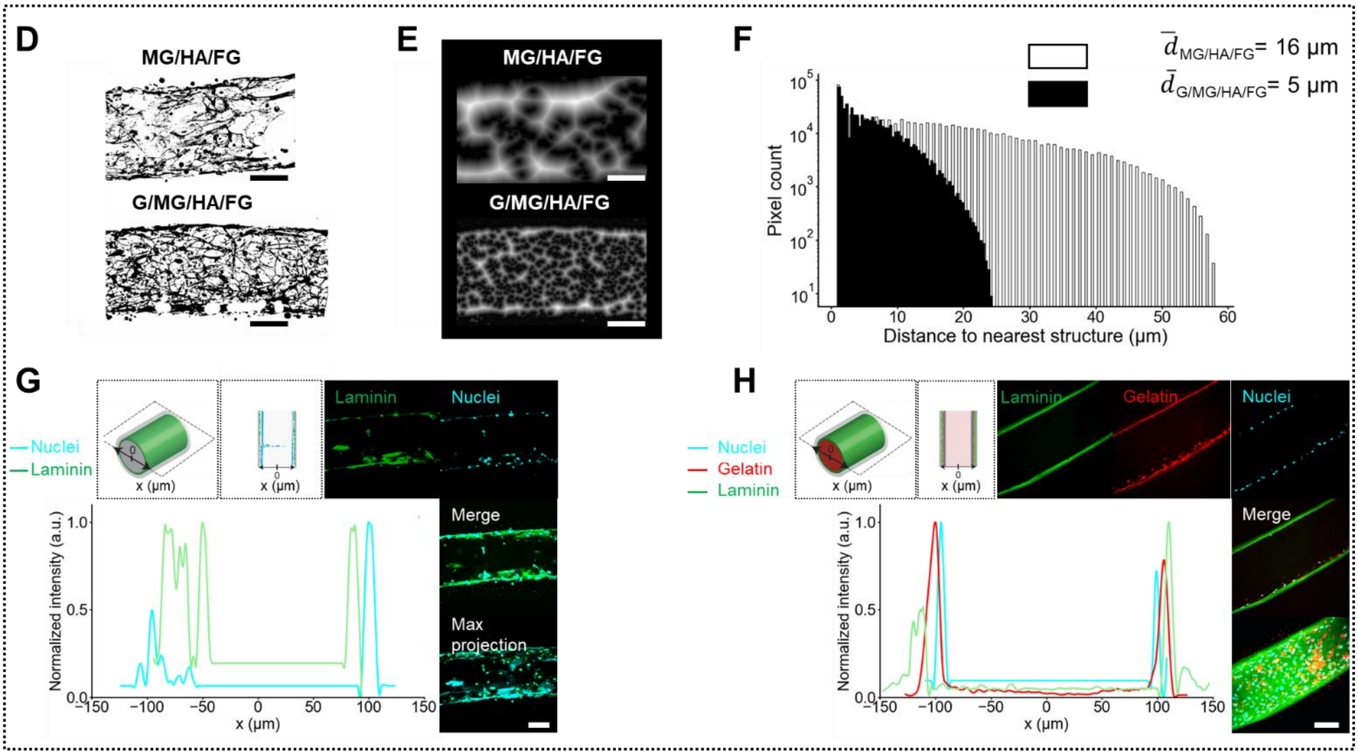

Supplementary Figure S4. **Matrix-driven HDLEC organization in tubular constructs**

(A) Brightfield images of HDLEC-laden artificial lymphatic endothelium formed in alginate-shelled tubes using various matrix compositions, cultured up to 7 days. Matrices were composed of 1-, 2-, 3-, or 4-component formulations based on gelatin (G), Matrigel (MG), hyaluronic acid (HA), and fibrinogen (FG). G and MG serve as scaffold-forming proteins [1–4], while HA and FG are bioactive factors known to influence lymphatic endothelial behavior [5, 6].

(a) 1-component matrices: With G alone, HDLECs remained rounded and sparse over time, with minimal spreading between day 2 and day 7. With MG alone, cells rapidly migrated and extended along the alginate-core interface by day 2, consistent with Matrigel coating of alginate [3, 4], forming aligned monolayers by day 7. HA alone failed to support any cell organization, lacking structural integrity. FG alone induced transient cord-like structures by day 2, which collapsed by day 7 due to insufficient mechanical support.

(b) 2-component matrices: HA/FG yielded diffuse, disorganized cell patterns. G/HA and G/FG supported limited spreading without alignment. MG/HA permitted partial elongation, and MG/FG promoted early alignment that was not maintained by day 7.

(c) 3-component matrices: MG/HA/FG induced dense, elongated cell assemblies reminiscent of endothelial cords, though full circumferential monolayers were not consistently observed.

(d) 4-component matrix (G/MG/HA/FG): Enabled robust and reproducible formation of peripheral HDLEC monolayers by day 7, indicative of stable artificial lymphatic endothelium.

These findings support distinct functional contributions of each component: FG promotes early nucleation and proliferation [6, 7], HA stabilizes nascent structures [5], MG promotes cellular polarization along interfaces [2–4], and G contributes to structural cohesion [1]. Only the 3- and 4-component matrices supported vessel-like morphologies after 7 days.

(B–C) Confocal maximum intensity projections of day-14 tubes stained for actin (magenta) and nuclei (cyan). MG/HA/FG constructs **(B)** exhibited fragmented cytoskeletal networks and uneven nuclear distribution. In contrast, G/MG/HA/FG constructs **(C)** showed continuous cortical actin and uniformly distributed nuclei, consistent with the formation of a continuous monolayer.

(D) Top-hat filtered binarized images derived from confocal actin filament images of entire representative tubes.

(E) Euclidean distance transform (EDT) maps generated from the processed confocal actin images shown in panel **D**.

(F) Histogram of nearest-neighbor distances quantifying the spatial organization of actin structures. Values were derived from Euclidean distance transform (EDT) maps (see panel **E**) for the two conditions: G/MG/HA/FG (solid black bars) and MG/HA/FG (white bars with black outlines). The distributions are plotted on a logarithmic scale and the mean spacing values $\bar{d}_{MG/HA/FG}$ and $\bar{d}_{G/MG/HA/FG}$ are indicated accordingly.

(G) Confocal cross-section of a MG/HA/FG construct at day 14, showing a 3D view of the equatorial plane of an alginate tube alongside a schematic 2D equatorial representation (laminin: green; nuclei: cyan). Individual fluorescence images of laminin, nuclei, and merged signals are presented, with corresponding fluorescence intensity profiles plotted along the x-axis.

(H) Confocal cross-section of a G/MG/HA/FG construct at day 14, showing a schematic 2D equatorial representation (laminin: green; gelatin: red; nuclei: cyan) followed by fluorescence images of laminin, nuclei, gelatin, and merged composite. Laminin localizes at the outer alginate interface, gelatin occupies the intermediate zone, and HDLECs form a continuous inner lining. Intensity profiles along the x-axis confirm spatial segregation of laminin, nuclei, and gelatin, indicating the formation of a stratified tubular architecture.

Scale bars: 200 µm **(A)**, 100 µm **(B–E)**, 50 µm **(G–H)**.

Supplementary Table S1. Morphological progression of lymphatic cell assemblies within tubular alginate constructs combining various natural ECM-derived protein components (gelatin, Matrigel, hyaluronic acid, and fibrinogen) at defined concentrations (w/v or v/v in total)
Lymphatic endothelial cells were encapsulated at 10^6 cells/mL and monitored by brightfield microscopy at Day 2 and Day 7. While most protein combinations yield limited morphological definition over time, specific 3-component and 4-component formulations—MG/HA/FG and G/MG/HA/FG—support the emergence of hollow-like lymphatic self-organized tubular structures after 7 days of culture.

Formulation	% Composition (in w/v or in v/v)	Day	Observed Outcome
G	Gelatin 2%	Day 2	Sparse distribution of individual cells
		Day 7	Low cell density; no visible tubular structures
MG	Matrigel 30%	Day 2	Evenly distributed cells across the gel
		Day 7	Partial linear alignment; no continuous structures
HA	Hyaluronic acid 0.2%	Day 2	Scattered, isolated cells
		Day 7	Diffuse signal; no visible organization
FG	Fibrinogen 0.2%	Day 2	Dispersed cells; weak structural presence
		Day 7	Reduced signal intensity; no tubular features
HA/FG	HA 0.2%/FG 0.2%	Day 2	Few grouped cells; unclear patterns
		Day 7	Isolated patches; no continuous structures
G/HA	G 2%/HA 0.2%	Day 2	Local clustering and partial alignment
		Day 7	Short tubular segments visible
G/FG	G 2%/FG 0.2%	Day 2	Thin, aligned lymphatic cell structures observed
		Day 7	Elongated lymphatic cell structures with visible continuity
MG/HA	MG 30%/HA 0.2%	Day 2	Moderately dispersed cell pattern
		Day 7	Sparse cellular distribution; no visible organization
MG/FG	MG 30%/FG 0.2%	Day 2	Initial arrangements of lymphatic cell structures
		Day 7	Outlined hollow-like tubular structures visible across the image
G/HA/FG	G 2%/HA 0.2%/FG 0.2%	Day 2	Elongated cell groupings with limited extension
		Day 7	Extended lymphatic cell structures with partial branching
MG/HA/FG	MG 30%/HA 0.2%/FG 0.2%	Day 2	Oriented cellular alignments observed
		Day 7	Outlined hollow-like tubular structures visible across the image
G/MG/HA/FG	G 2%/ MG 30%/HA 0.2%/FG 0.2%	Day 2	Continuous aligned structures spanning across the gel
		Day 7	Outlined hollow-like tubular networks distributed throughout the gel

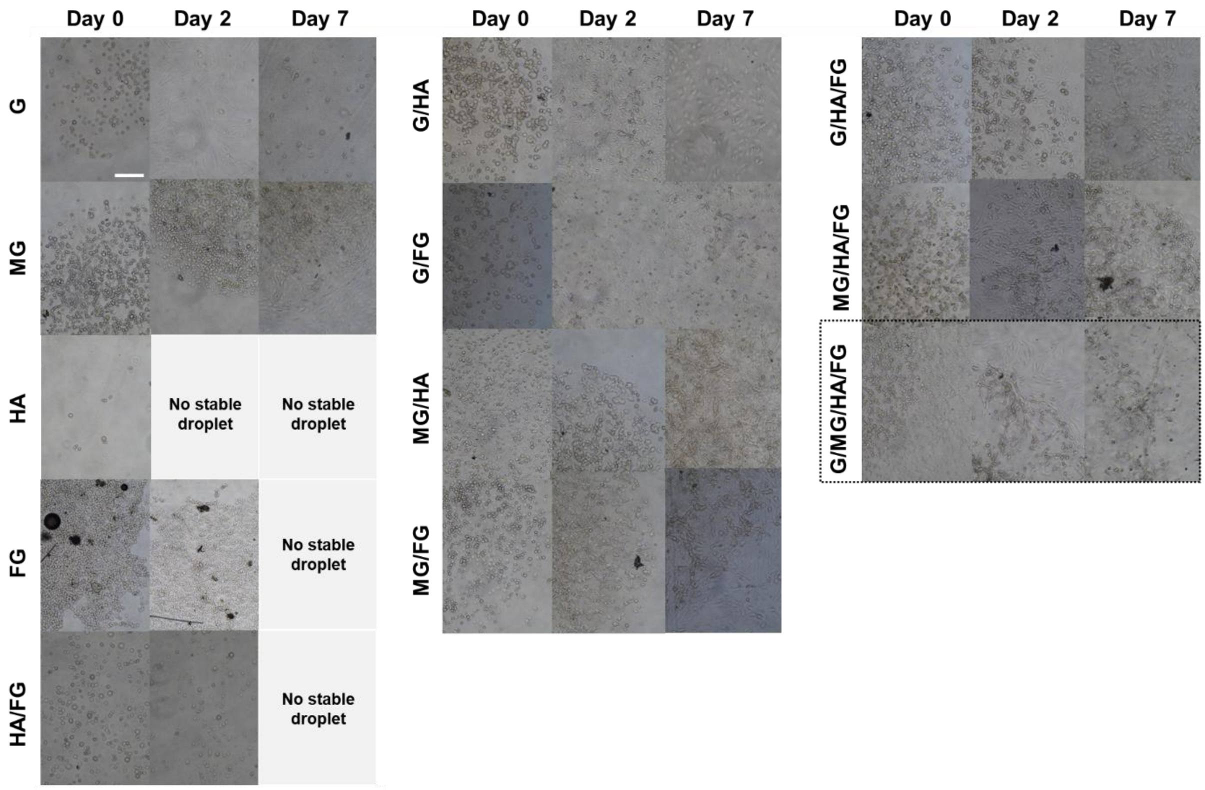

Supplementary Figure S5. Matrix-driven HDLEC organization in spherical domes

Brightfield and fluorescence images of HDLEC-laden droplets containing the same 1-, 2-, 3-, and 4-component formulations tested in Supplementary Figure S4 but polymerized without alginate to assess HDLEC behavior in unconfined environments. Actin (magenta) and nuclei (cyan) are shown at day 2 and day 7.

In 1-component droplets, gelatin (G) and Matrigel (MG) supported limited cell retention, with MG promoting early spreading and peripheral organization [1–4]. Hyaluronic acid (HA) and fibrinogen (FG) alone failed to maintain structural integrity and rapidly dispersed in the medium.

Among 2-component formulations, MG/HA and MG/FG supported partial cord formation, while other combinations remained poorly organized. The 3-component MG/HA/FG matrix led to compact multicellular assemblies by day 7. The 4-component G/MG/HA/FG matrix resulted in the most cohesive structures, forming organized vessel-like cords even in the absence of confinement.

Together, these results emphasize the importance of combining scaffold-forming [1–4] and bioactive components [5–7]. In unconfined environments, MG promotes early spatial organization, while HA and FG contribute to structural stabilization and morphogenesis. The 4-component formulation emerged as the most effective matrix to guide HDLECs into stable, self-organized vascular structures.

Formulations that failed to generate stable droplets are indicated as “no stable droplet”.

Scale bar: 200 µm.

Supplementary Table S2. Morphological progression of lymphatic cell assemblies within spherical domes combining natural ECM-derived protein components at defined concentrations (w/v or v/v in total)

Lymphatic cells were encapsulated at 10^6 cells/mL and monitored by brightfield microscopy at Day 2 and Day 7. Early culture stages revealed scattered or loosely grouped rounded cells, while several 3-component and 4-component formulations, notably G/MG/HA/FG, promoted the formation of compact lymphatic cell structures suggestive of cord-like network assembly.

Formulation	% Composition (in w/v or in v/v)	Day	Observed Outcome
G	Gelatin 2%	Day 2	Moderate cell clustering; spherical cellular groupings observed
		Day 7	Low cell density; partial disaggregation of cell clusters
MG	Matrigel 30%	Day 2	Centrally located cellular groupings; compact cell structures involved in lymphatic cord-like network assembly
		Day 7	Persistent compact cell structures; reduced peripheral cells
HA	Hyaluronic acid 0.2%	Day 2	Image not available for this condition at this time point
		Day 7	Image not available for this condition at this time point
FG	Fibrinogen 0.2%	Day 2	Scattered rounded cells; minimal cellular grouping
		Day 7	Image not available for this condition at this time point
HA/FG	HA 0.2%/FG 0.2%	Day 2	Small cellular groupings; unevenly distributed
		Day 7	Image not available for this condition at this time point
G/HA	G 2%/HA 0.2%	Day 2	Formation of isolated cell structures involved in lymphatic cord-like network assembly
		Day 7	Compact isolated cell structures with no observable continuity between them
G/FG	G 2%/FG 0.2%	Day 2	Dense small aggregates of cells observed
		Day 7	Presence of compact round cell structures
MG/HA	MG 30%/HA 0.2%	Day 2	Centrally located cellular groupings with homogeneous distribution
		Day 7	Increased proximity of cells; persistent multicellular groupings
MG/FG	MG 30%/FG 0.2%	Day 2	Cell structures with central condensation observed
		Day 7	Well-defined, persistent cell structures involved in lymphatic cord-like network assembly
G/HA/FG	G 2%/HA 0.2%/FG 0.2%	Day 2	Loose cellular groupings scattered across the matrix
		Day 7	More compact cell clusters; low structural regularity
MG/HA/FG	MG 30%/HA 0.2%/FG 0.2%	Day 2	Prominent compact cell structures involved in lymphatic cord-like network assembly
		Day 7	Stable, well-defined cell structures involved in lymphatic cord-like network assembly
G/MG/HA/FG	G 2%/MG 30%/HA 0.2%/FG 0.2%	Day 2	Numerous well-separated cell structures involved in lymphatic cord-like network assembly
		Day 7	Outlined, stable cell structures involved in lymphatic cord-like network assembly (highlighted in the figure)

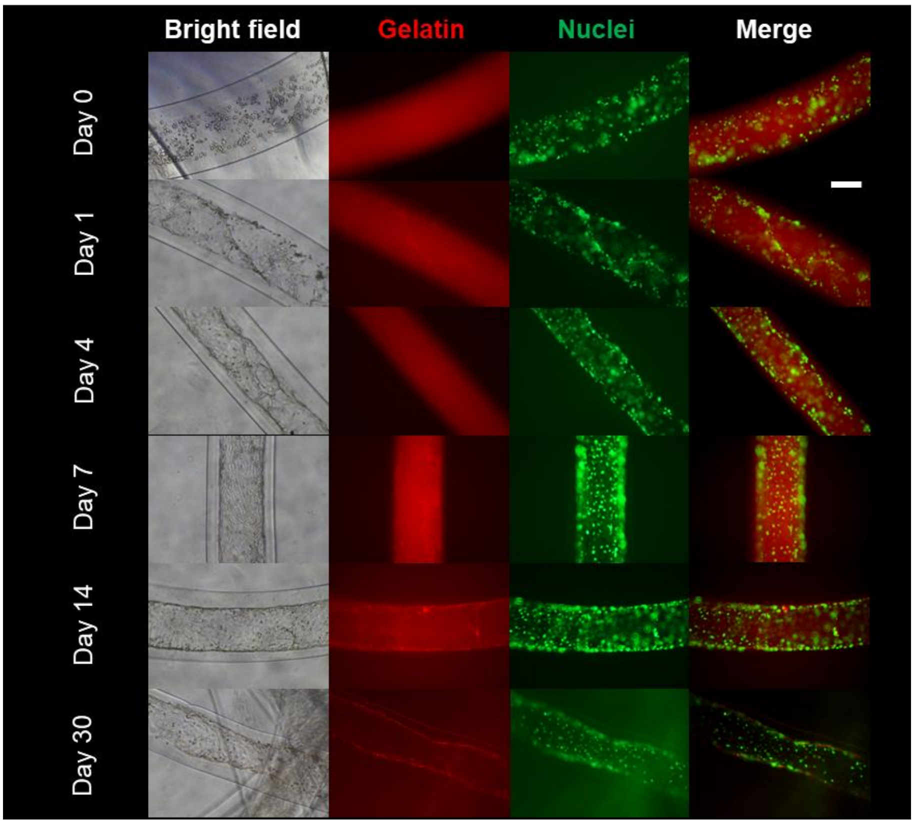

Supplementary Figure S6. Time-course monitoring of gelatin remodeling and HDLEC organization within alginate-core constructs (day 0 to day 30)
Representative brightfield and fluorescence images of HDLEC-seeded constructs at days 0, 1, 4, 7, 14, and 30. Nuclei (green) correspond to HDLECs stably expressing H2B-GFP. The hydrogel core contains rhodamine-labeled gelatin (G_{rh}; red) as part of the G_{rh}/MG/HA/FG matrix. Fluorescence and structural changes over time reflect progressive cell organization and gelatin remodeling.
Scale bar: 100 µm**.**

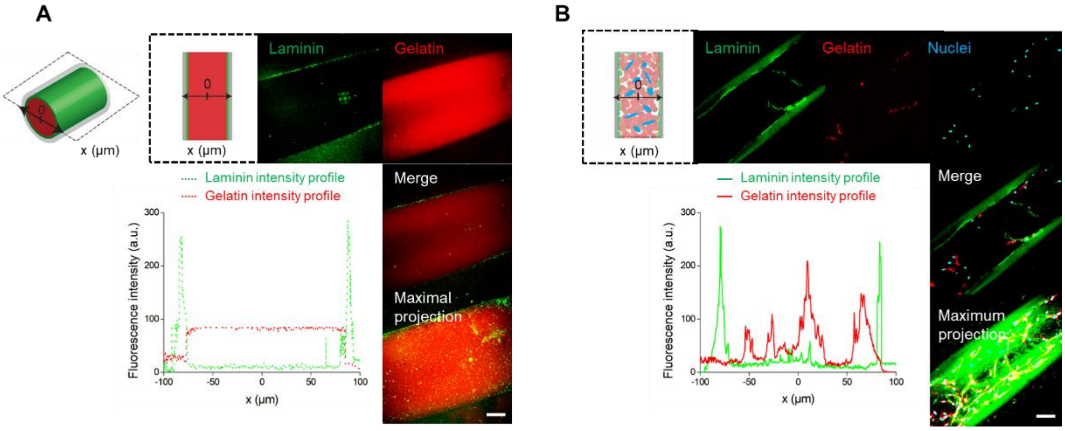

Supplementary Figure S7. **Matrix component distribution at day 1 with and without cells**
(A) Cell-free constructs show uniform gelatin distribution (red) and peripheral laminin (green) coating the alginate wall, consistent with spontaneous Matrigel anchorage [2–4].
(B) HDLEC-laden constructs show an initial matrix organization similar to that of cell-free controls, with no visible remodeling at this early stage.
Scale bars: 50 μm**.**

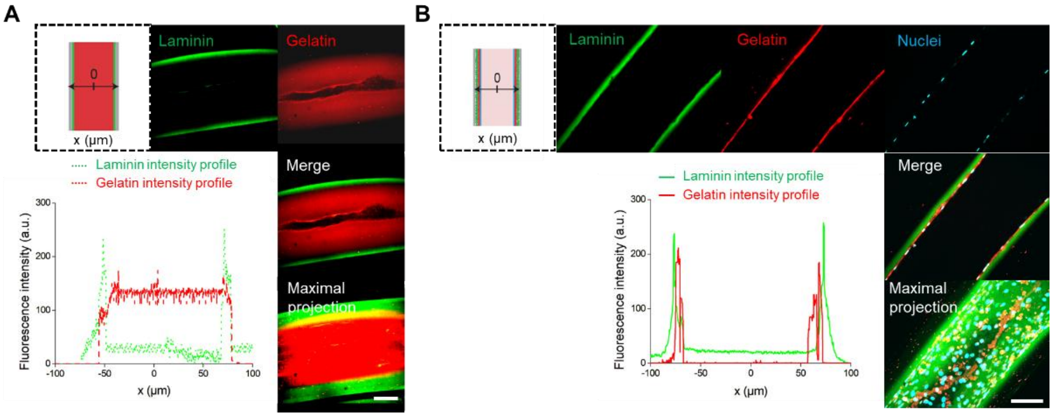

Supplementary Figure S8. Matrix component distribution at day 14 with and without cells

(A) Cell-free constructs maintain a largely unchanged matrix organization, with uniform gelatin distribution (red) and peripheral laminin (green) coating the alginate wall, consistent with spontaneous Matrigel anchorage [2–4].

(B) In HDLEC-laden constructs, matrix organization is altered relative to the cell-free condition, with evidence of cell-associated remodeling and progressive spatial redistribution of matrix components at the tube wall.

Scale bars: 50 µm**.**

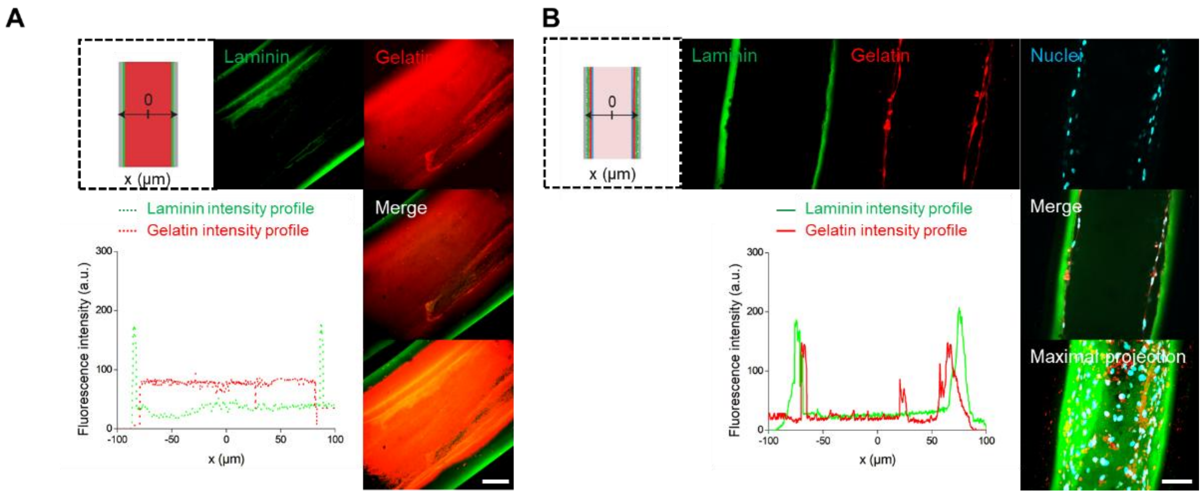

Supplementary Figure S9. Matrix component distribution at day 30 with and without cells
(A) Cell-free constructs retain the initial matrix organization, with persistent uniform gelatin distribution (red) and peripheral laminin (green) coating the alginate wall**.**
(B) In HDLEC-laden constructs, the matrix is markedly reorganized by day 30, consistent with long-term cell-mediated remodeling and stratification at the tube wall.
Scale bars: 50 µm**.**

Supplementary Table S3. **Statistical analysis of PHH3-positive cell percentages across the culture period (Days 1 to 30), corresponding to Fig. 2B**

Mitotic activity was assessed by quantifying the percentage of PHH3-positive nuclei at each culture time point. One-way ANOVA followed by Tukey's post hoc multiple comparisons test was used to identify statistically significant differences between all pairs of time points. The table provides the adjusted p-values along with corresponding levels of significance (**** p < 0.0001; *** p < 0.001; ** p < 0.01; * p < 0.05; n.s. = not significant).

To optimize readability of the graph (**Fig. 2B**), only comparisons with p > 0.05 ("n.s.") are explicitly shown. All significant comparisons are listed in this table for comprehensive reference.

Comparison	p-value	Significance
Day 1 vs Day 14	<1e-16	****
Day 1 vs Day 21	<1e-16	****
Day 1 vs Day 3	<1e-16	****
Day 1 vs Day 30	<1e-16	****
Day 1 vs Day 5	<1e-16	****
Day 1 vs Day 7	0.0004	***
Day 14 vs Day 21	0.8258	n.s.
Day 14 vs Day 3	<1e-16	****
Day 14 vs Day 30	0.1148	n.s.
Day 14 vs Day 5	<1e-16	****
Day 14 vs Day 7	0.9113	n.s.
Day 21 vs Day 3	<1e-16	****
Day 21 vs Day 30	0.7812	n.s.
Day 21 vs Day 5	<1e-16	****
Day 21 vs Day 7	0.1770	n.s.
Day 3 vs Day 30	<1e-16	****
Day 3 vs Day 5	<1e-16	****
Day 3 vs Day 7	<1e-16	****
Day 30 vs Day 5	<1e-16	****
Day 30 vs Day 7	0.0062	**
Day 5 vs Day 7	<1e-16	****

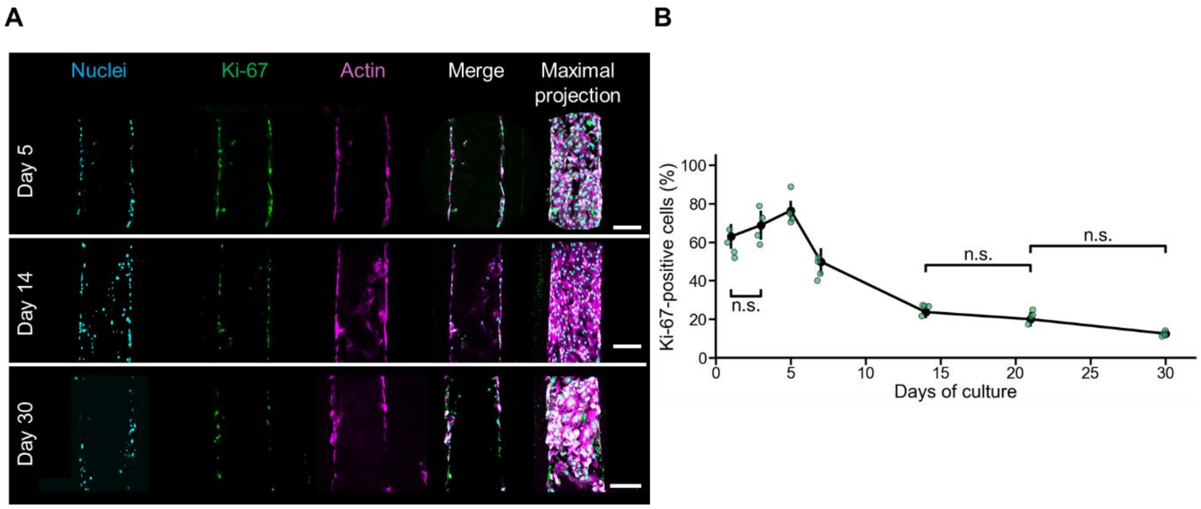

Supplementary Figure S10. Confocal imaging and quantitative analysis of Ki-67 expression in 3D lymphatic endothelial constructs

(A) Confocal equatorial images showing immunostaining for nuclei (cyan), Ki-67 (green), and actin (magenta) at day 5, day 14, and day 30. Merged equatorial as well as maximal projection views highlight spatial localization of Ki-67-positive cells and changes in signal density.

(B) Scatter plot displaying individual values for the percentage of Ki-67-positive nuclei over time. The proportion of proliferating cells peaked at day 5 (>75%) and progressively declined below 15% by day 30, indicating a transition to quiescence. Data are shown as mean ± SD. One-way ANOVA with Tukey's post hoc test was performed. Only comparisons with $p > 0.05$ are displayed (labeled as "n.s."); all others were statistically significant ($p < 0.01$, not shown).

N ≥ 3 constructs per time point.

Scale bars: 100 µm.

Supplementary Table S4. **Statistical summary of Ki-67-positive cell percentages across the culture timeline (Days 1 to 30), corresponding to Fig. S10B**

To analyze the temporal dynamics of proliferating cells, the percentage of Ki-67-positive nuclei was quantified at each time point. Statistical significance was evaluated using one-way ANOVA followed by Tukey's post-hoc test, allowing pairwise comparisons between all days. The table lists the adjusted p-values along with their respective significance indicators (**** $p < 0.0001$; *** $p < 0.001$; ** $p < 0.01$; * $p < 0.05$; n.s. = not significant).

Comparison	p-value	Significance
Day 1 vs Day 3	0.2777	n.s.
Day 1 vs Day 5	<1e-16	****
Day 1 vs Day 7	<1e-16	****
Day 1 vs Day 14	<1e-16	****
Day 1 vs Day 21	<1e-16	****
Day 1 vs Day 30	<1e-16	****
Day 3 vs Day 5	0.0589	n.s.
Day 3 vs Day 7	<1e-16	****
Day 3 vs Day 14	<1e-16	****
Day 3 vs Day 21	<1e-16	****
Day 3 vs Day 30	<1e-16	****
Day 5 vs Day 7	<1e-16	****
Day 5 vs Day 14	<1e-16	****
Day 5 vs Day 21	<1e-16	****
Day 5 vs Day 30	<1e-16	****
Day 7 vs Day 14	<1e-16	****
Day 7 vs Day 21	<1e-16	****
Day 7 vs Day 30	<1e-16	****
Day 14 vs Day 21	0.8021	n.s.
Day 14 vs Day 30	0.004	**
Day 21 vs Day 30	0.0507	n.s.

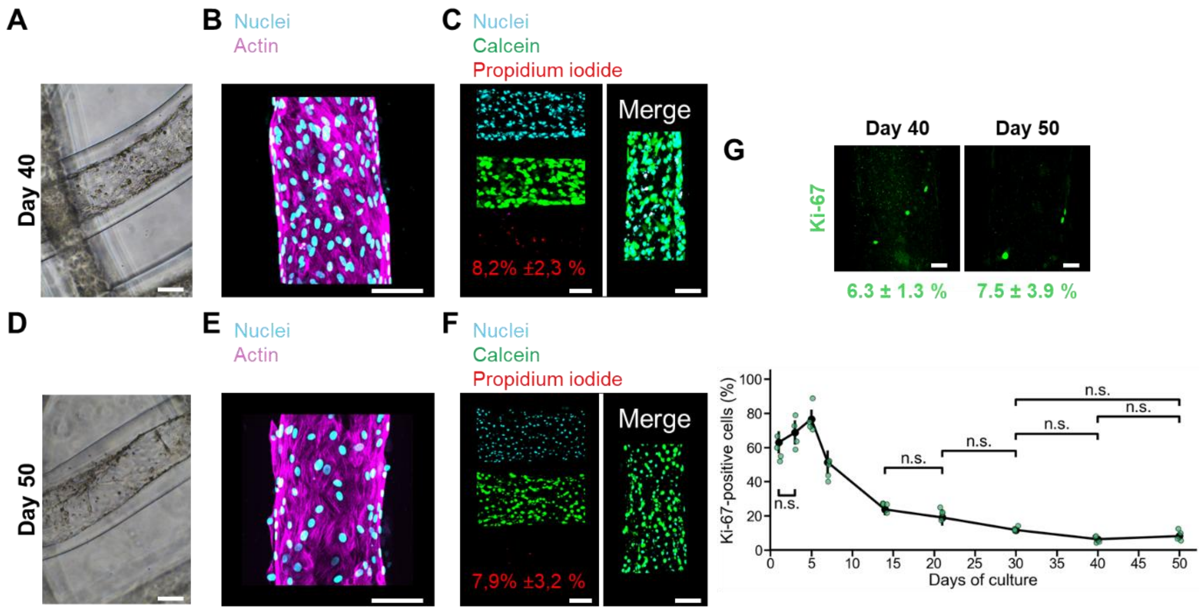

Supplementary Figure S11. Extended analysis of structural stability, viability, and proliferative activity in artificial 3D lymphatic endothelium at days 40 and 50 under static culture conditions

(A, B) Brightfield images of constructs at days 40 **(A)** and 50 **(B)**, showing maintained tubular morphology and cell density.

(D, E) Confocal maximum intensity projections stained for nuclei (cyan) and actin (magenta) at days 40 **(D)** and 50 **(E)**, revealing persistent cortical actin organization and regular nuclear distribution.

(C, F) Viability assays at days 40 **(C)** and 50 **(F)**, with nuclei (cyan), live cells (green), and dead cells (red) staining. Quantification indicates low and stable percentages of dead cells (~8%) at both time points.

(G) Ki-67 immunostaining at days 40 and 50 showing minimal proliferative activity. Dot plot showing the percentage of Ki-67-positive nuclei over time. A sharp decrease in proliferation is observed from day 7 onward, dropping below 30% at day 14 and stabilizing under 20% from day 21. Data are shown as mean ± SD. One-way ANOVA followed by Tukey's post hoc test was performed. Only pairwise comparisons with $p > 0.05$ are displayed (denoted as "n.s."). All other comparisons were statistically significant ($p < 0.01$, not shown). N ≥ 3 tubes for viability and Ki-67 analysis.

Scale bars: 100 µm **(A–F)**, 50 µm **(G)**.

Supplementary Table S5. Statistical analysis of Ki-67-positive cell percentages across culture time from Day 1 to Day 50, related to Fig. S11G

Cell proliferation dynamics were quantified by assessing the percentage of Ki-67-positive nuclei at each time point. One-way ANOVA followed by Tukey's multiple comparisons test was used to evaluate statistical differences between all pairs of time points. The table reports adjusted p-values and corresponding significance levels (**** $p < 0.0001$; *** $p < 0.001$; ** $p < 0.01$; * $p < 0.05$; n.s. = not significant).

Comparison	p-value	Significance
Day 1 vs Day 14	<1e-16	****
Day 1 vs Day 21	<1e-16	****
Day 1 vs Day 3	0.2267	n.s.
Day 1 vs Day 30	<1e-16	****
Day 1 vs Day 40	<1e-16	****
Day 1 vs Day 5	<1e-16	****
Day 1 vs Day 50	<1e-16	****
Day 1 vs Day 7	1.000e-04	***
Day 14 vs Day 21	0.5133	n.s.
Day 14 vs Day 3	<1e-16	****
Day 14 vs Day 30	0.004	**
Day 14 vs Day 40	<1e-16	****
Day 14 vs Day 5	<1e-16	****
Day 14 vs Day 50	<1e-16	****
Day 14 vs Day 7	<1e-16	****
Day 21 vs Day 3	<1e-16	****
Day 21 vs Day 30	0.0543	n.s.
Day 21 vs Day 40	<1e-16	****
Day 21 vs Day 5	<1e-16	****
Day 21 vs Day 50	0.0004	***
Day 21 vs Day 7	<1e-16	****
Day 3 vs Day 30	<1e-16	****
Day 3 vs Day 40	<1e-16	****
Day 3 vs Day 5	0.0589	n.s.
Day 3 vs Day 50	<1e-16	****
Day 3 vs Day 7	<1e-16	****
Day 30 vs Day 40	0.3295	n.s.
Day 30 vs Day 5	<1e-16	****
Day 30 vs Day 50	0.8225	n.s.
Day 30 vs Day 7	<1e-16	****
Day 40 vs Day 5	<1e-16	****
Day 40 vs Day 50	0.9969	n.s.
Day 40 vs Day 7	<1e-16	****
Day 5 vs Day 50	<1e-16	****
Day 5 vs Day 7	<1e-16	****
Day 50 vs Day 7	<1e-16	****

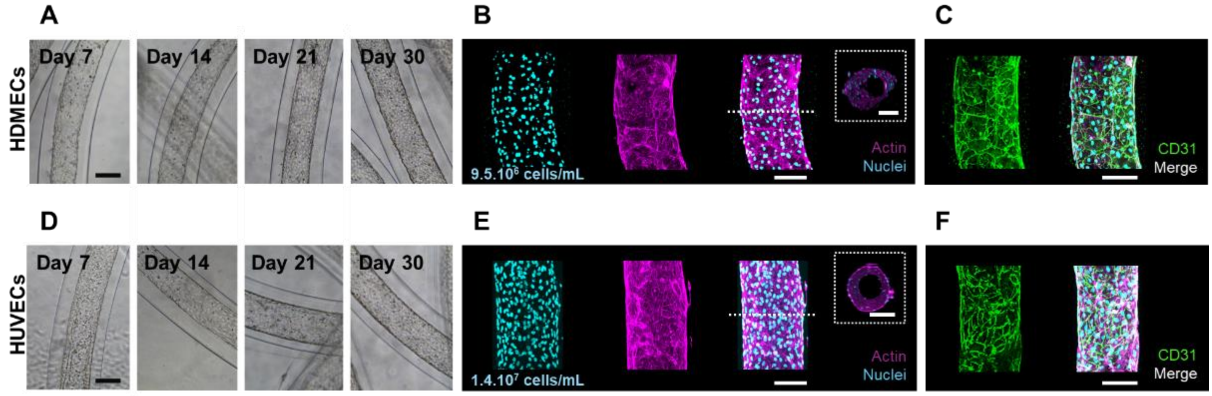

Supplementary Figure S12. Sustained maintenance of lineage-specific 3D endothelial monolayers in the optimized hydrogel matrix

(A, D) Brightfield images of HDMEC-based **(A)** and HUVEC-based **(D)** constructs at days 7, 14, 21, and 30 of culture. Both cell types maintain a dense and morphologically stable monolayer over time. The progressive darkening of the tube walls suggests increased cellular compaction compared with HDLEC-based structures (see **Fig. 3A**).

(B, E) Confocal maximum-intensity projections of HDMEC-based **(B)** and HUVEC-based **(E)** constructs at day 30, showing nuclei (cyan), actin (magenta), and merged images. Insets show 3D reconstructions with selected viewing angles, confirming the formation of continuous monolayers fully covering the luminal surface. Nuclear densities are higher than in HDLEC-based constructs, with approximately 9.6 × 10^6 cells/mL for HDMECs and 1.4 × 10^7 cells/mL for HUVECs. N ≥ 3 independent extrusion batches were analyzed per condition.

(C, F) Confocal maximum-intensity projections of HDMEC-based **(C)** and HUVEC-based **(F)** constructs at day 30, showing CD31 (green) and merged images with nuclei and actin signals, confirming that blood microvascular endothelia establish similarly continuous $CD31^+$ junctional networks.

Scale bars: 100 µm.

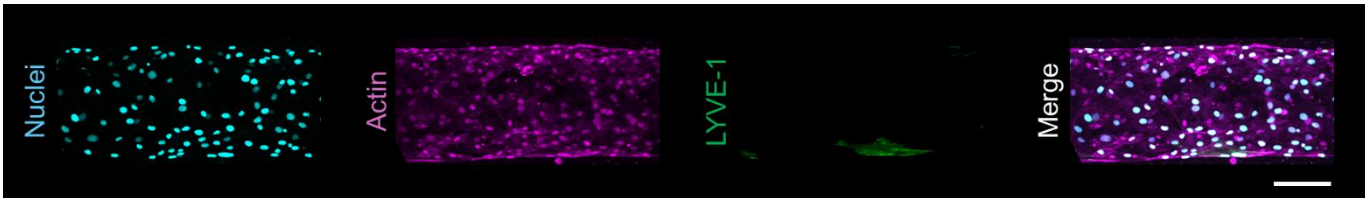

Supplementary Figure S13. LYVE-1 staining in HDMEC tubular constructs Confocal maximum-intensity projections of HDMEC-based tubular constructs at day 30 showing nuclei (cyan), F-actin (magenta), and LYVE-1 (green), and corresponding merged images. These images were acquired under the same imaging conditions as the HDLEC constructs shown in **Figure 3**. HDMEC tubes display sparse and weak LYVE-1 signal compared with HDLEC tubes, confirming the lineage-specific enrichment of LYVE-1 in lymphatic endothelial constructs.
Scale bars: 100 µm.

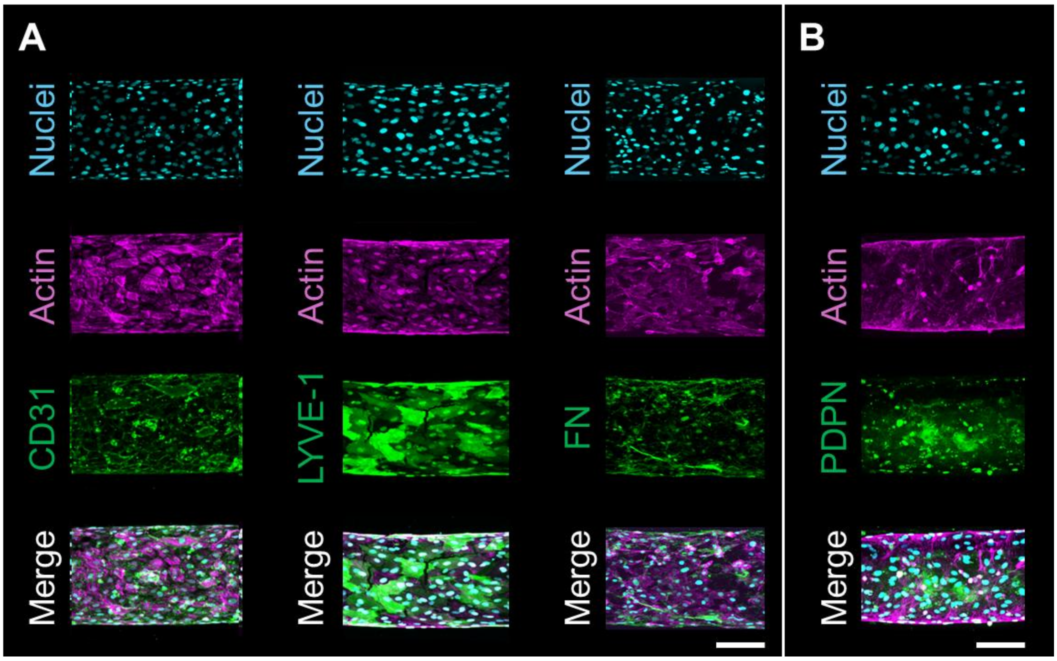

Supplementary Figure S14. Consolidation and persistence of lymphatic identity markers and ECM remodeling in HDLEC tubes

(A) Confocal maximum-intensity projections of HDLEC-based tubular constructs at day 14, showing nuclei (cyan), actin (magenta), and CD31, LYVE-1 or fibronectin (FN green), with corresponding merged images.

(B) Confocal maximum-intensity projections of HDLEC-based constructs stained for PDPN (green) at day 30 together with nuclei and actin. These data indicate that PDPN expression is maintained at least until day 30 of culture.

Scale bars: 100 µm.

A

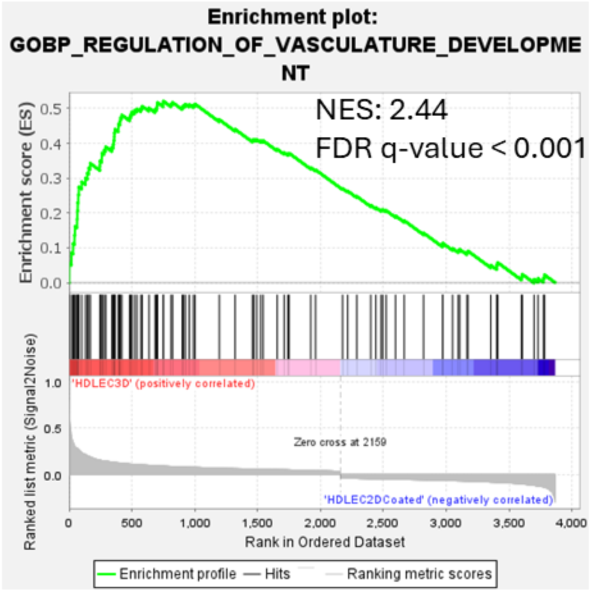

B

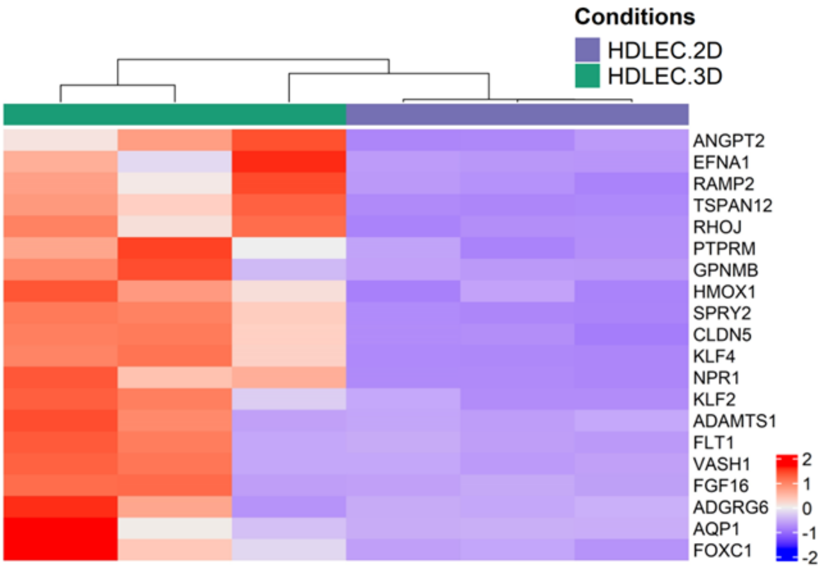

Supplementary Figure S15. Transcriptomic enrichment of regulation of vasculature development-related genes in HDLEC 3D when compared to HDLEC 2D

(A) GSEA enrichment plot for regulation of vasculature development signatures. Normalized Enrichment Score (NES) and False Discovery Rate (FDR) q-value are indicated on the plot.

(B) Heatmap of the top 20 core enriched genes across the signatures presented in **(A)**.

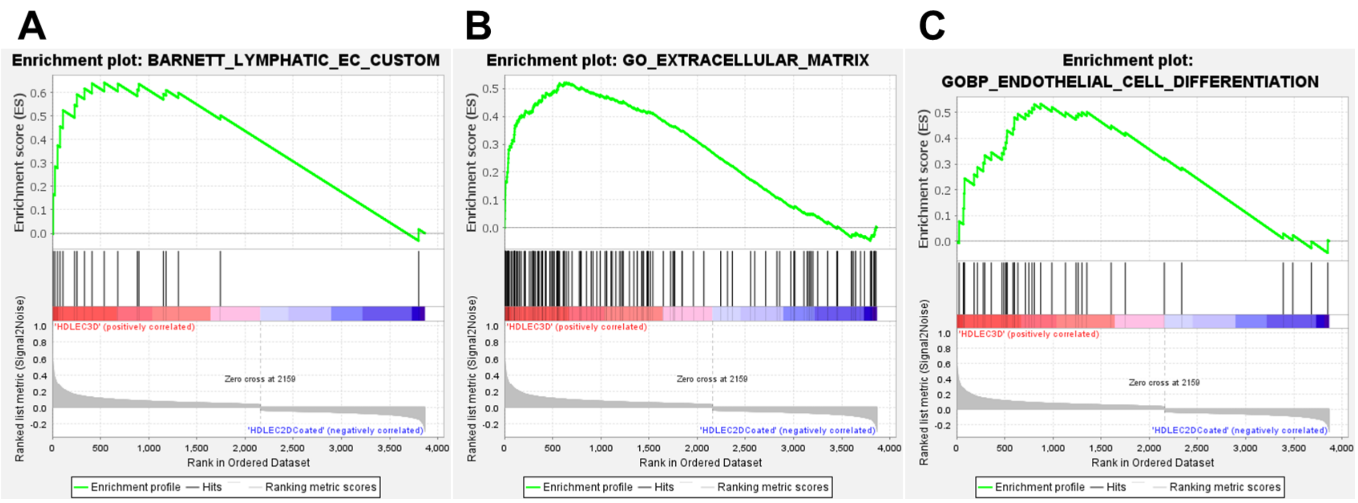

Supplementary Figure S16. **GSEA enrichment plots for HDLEC 3D versus HDLEC 2D**

(A) Enrichment of a curated lymphatic endothelial cell signature derived from single-cell RNA-seq data ([8]; 101 genes selected on the basis of logFC > 2, adjusted p-value below the numerical precision threshold, pct_nz_group > 10, and pct_nz_group/pct_nz_reference > 2) in HDLEC 3D compared with HDLEC 2D (NES = 2.11, FDR q-value < 0.001).

(B) Enrichment of the extracellular matrix organization gene set (NES = 2.59, FDR q-value < 0.001).

(C) Enrichment of the endothelial cell differentiation gene set (NES = 2.06, FDR q-value = 0.006). Each plot displays the running enrichment score across the ranked gene list; the barcode indicates the position of gene set members. The leading-edge subset represents the core genes driving the enrichment signal.

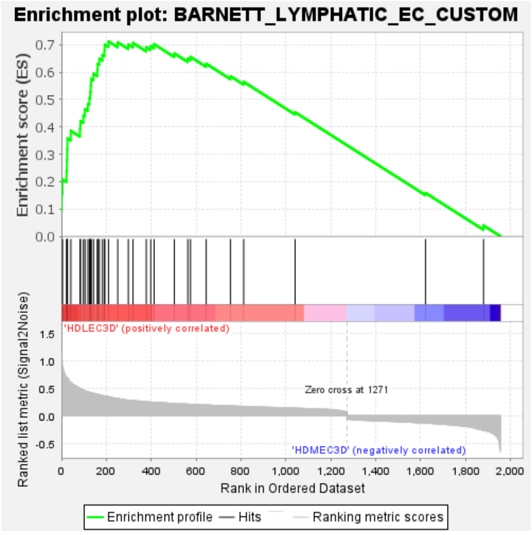

Supplementary Figure S17. **GSEA enrichment plot for HDLEC 3D versus HDMEC 3D**
Enrichment of the curated lymphatic endothelial cell signature [8] in HDLEC 3D compared with HDMEC 3D (NES = 2.77, FDR q-value < 0.001).
Plot format as described in Supplementary Figure S16.

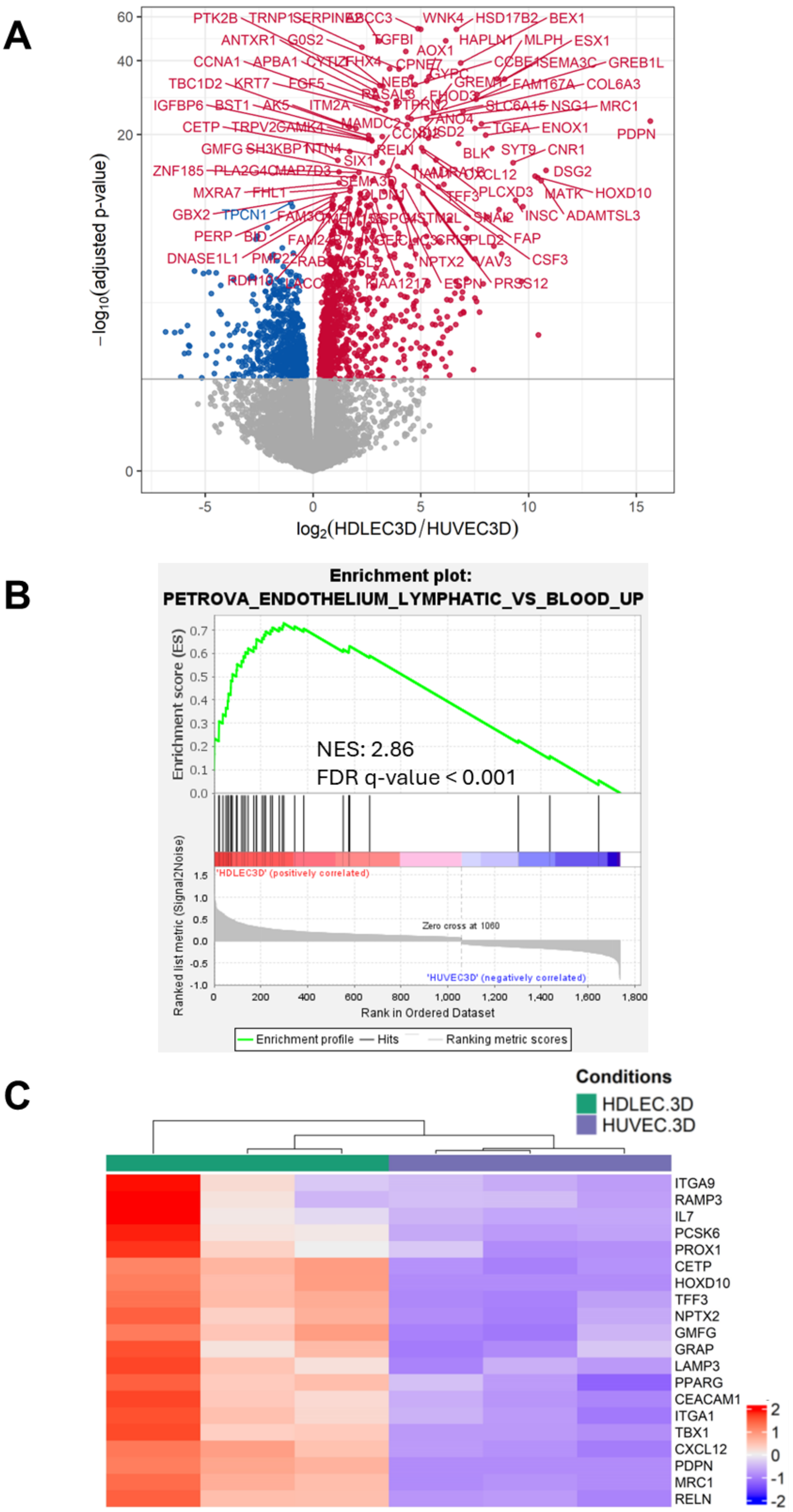

Supplementary Figure S18. Transcriptomic profiling of 3D lymphatic (HDLECs) and blood endothelial (HUVECs) tubes

(A) Volcano plot of differentially expressed genes between HDLEC 3D and HUVEC 3D. Significantly upregulated and downregulated genes are represented by red and blue dots, respectively.

(B) GSEA enrichment plot according to PETROVA_ENDOTHELIUM_LYMPHATIC_VS_BLOOD_UP gene signature. Normalized Enrichment Score (NES) and False Discovery Rate (FDR) q-value are indicated on the plot.

(C) Heatmap of the top 20 core enriched genes across the signature presented in **(B)**.

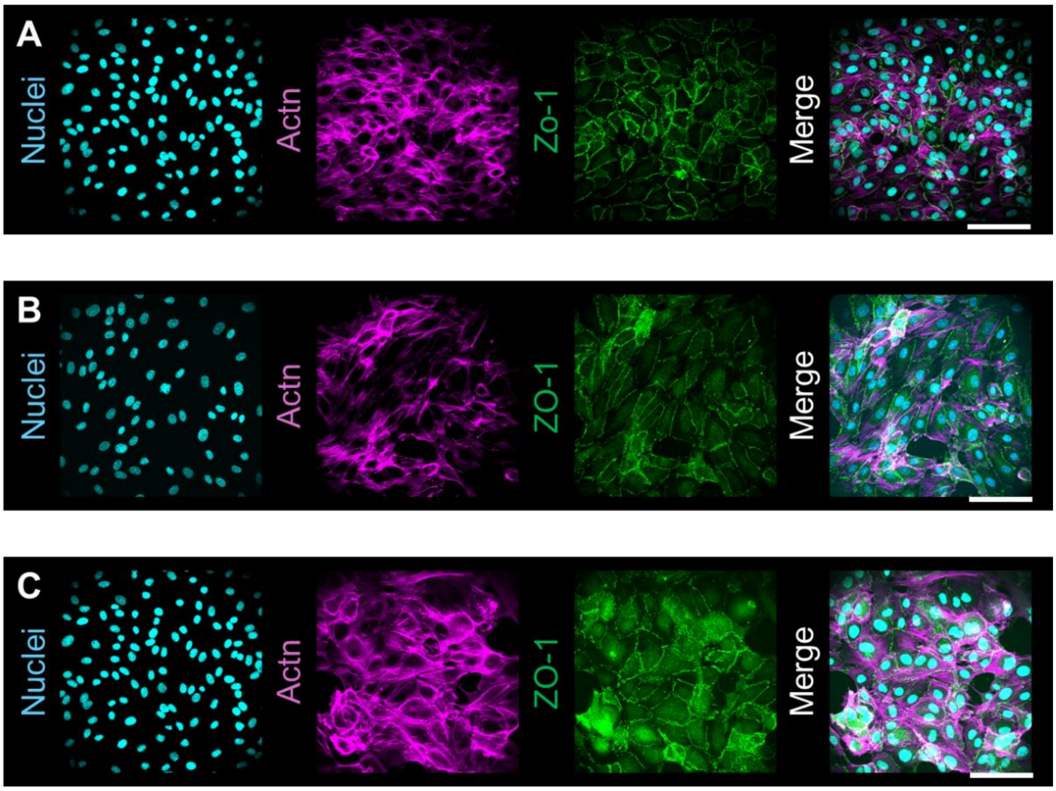

Supplementary Figure S19. **ZO-1 expression in 2D cultures of endothelial cells**
(A–C) HDLECs **(A)**, HDMECs **(B),** and HUVECs **(C)** cultured on 2D substrates all express ZO-1 at intercellular junctions under identical conditions. This confirms that the reduced ZO-1 signal in HDLEC-based 3D constructs reflects differences in 3D junctional organization rather than loss of protein expression.
Scale bars: 100 µm.

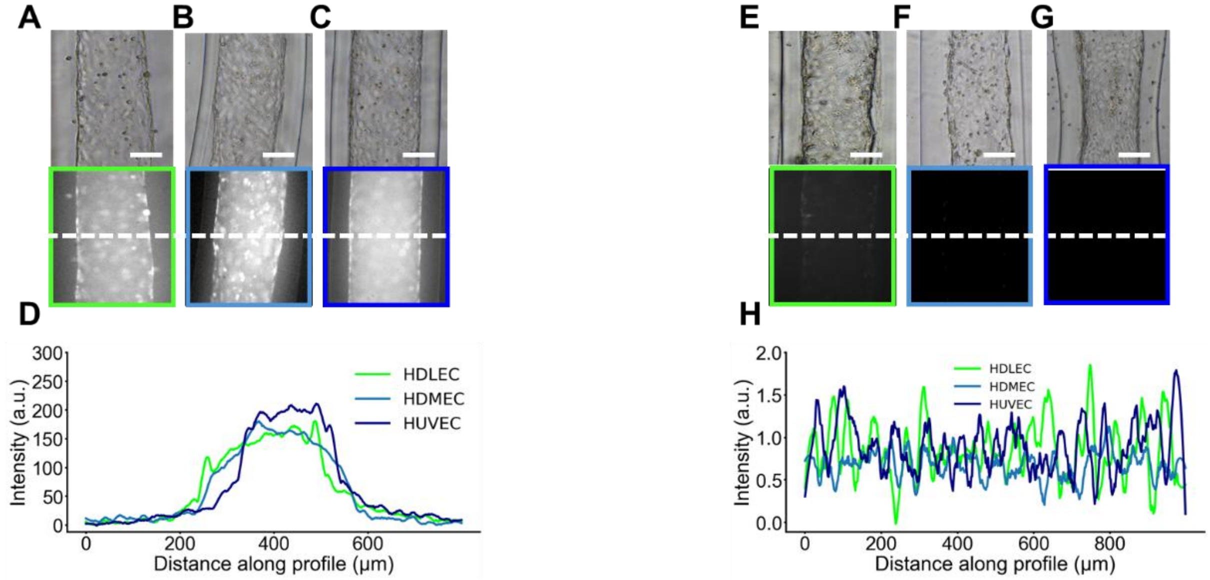

Supplementary Figure S20. Size-dependent permeability of endothelial constructs

(A–D) Permeability assay using 3 kDa rhodamine-dextran. Constructs composed of HDLECs **(A)**, HDMECs **(B)**, and HUVECs **(C)** were incubated with 3 kDa fluorescent tracer. Fluorescence imaging revealed tracer accumulation in the lumen of all constructs, indicating permeation across the alginate shell and endothelial layers. Panel D shows intensity profiles across tube cross-sections, confirming comparable tracer penetration in all conditions.

(E–H) Permeability assay using 500 kDa rhodamine-dextran. Constructs composed of HDLECs **(E)**, HDMECs **(F)**, and HUVECs **(G)** were incubated with high-molecular-weight tracer. No luminal fluorescence signal was detected in any condition, as shown in intensity profiles **(H)**, confirming that 500 kDa dextran is effectively excluded by both the surrounding alginate layer and the endothelial barrier [9].

Scale bars: 100 µm.

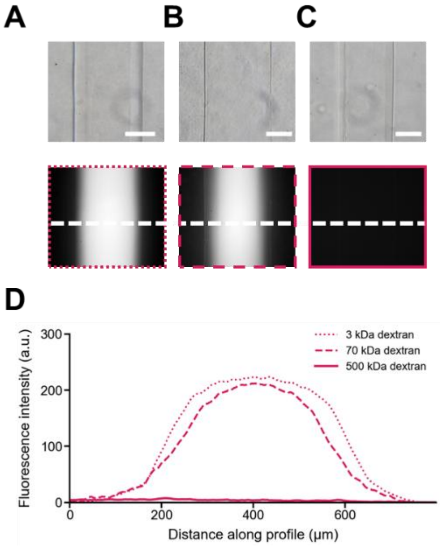

Supplementary Figure S21. Size-dependent diffusion of fluorescent dextrans in acellular hydrogel tubes

(A–C) Brightfield (top) and fluorescence images (bottom) of acellular G/MG/HA/FG tubes incubated with rhodamine-conjugated dextrans of increasing molecular weight: 3 kDa **(A)**, 70 kDa **(B)**, and 500 kDa **(C)**. The dashed white line indicates the line ROI used to extract fluorescence intensity profiles across the tube cross-section. **(D)** Fluorescence intensity profiles measured along the ROI shown in **A–C**. Both 3 kDa and 70 kDa dextrans readily equilibrate between the external medium and the tube lumen, whereas the 500 kDa dextran shows minimal luminal penetration, confirming that the hydrogel scaffold does not constitute a diffusion barrier for tracers in the 3–70 kDa range.

Scale bars: 100 μm.

Supplementary datasets

Supplementary Dataset 1. Differential expression results — HDLEC 3D vs HDLEC 2D (Excel file)
Content: Differential gene expression analysis comparing HDLEC tubes cultured in 3D versus conventional 2D HDLEC monolayers.
Columns:
Gene: gene symbol (identifier).
baseMean: mean of normalized counts across all samples.
log2FoldChange: log2 fold change (HDLEC 3D / HDLEC 2D); positive values indicate higher expression in 3D.
lfcSE: standard error of log2FoldChange.
stat: Wald statistic.
pvalue: Wald test p-value.
padj: Benjamini–Hochberg adjusted p-value (FDR).
Number of rows: 14391 genes.

Supplementary Dataset 2. Differential expression results — HDLEC 3D vs HDMEC 3D (Excel file)
Content: Differential gene expression analysis comparing lymphatic (HDLEC) and blood microvascular (HDMEC) endothelial tubes cultured under matched 3D conditions.
Columns:
Gene: gene symbol (identifier).
baseMean: mean of normalized counts across all samples.
log2FoldChange: log2 fold change (HDLEC 3D / HDMEC 3D); positive values indicate higher expression in HDLEC 3D.
lfcSE: standard error of log2FoldChange.
stat: Wald statistic.
pvalue: Wald test p-value.
padj: Benjamini–Hochberg adjusted p-value (FDR).
Number of rows: 14183 genes.

Supplementary Dataset 3. Differential expression results — HDLEC 3D vs HUVEC 3D (Excel file)
Content: Differential gene expression analysis comparing lymphatic (HDLEC) and blood endothelial (HUVEC) tubes cultured under matched 3D conditions.
Columns:
Gene: gene symbol (identifier).
baseMean: mean of normalized counts across all samples.
log2FoldChange: log2 fold change (HDLEC 3D / HUVEC 3D); positive values indicate higher expression in HDLEC 3D.
lfcSE: standard error of log2FoldChange.
stat: Wald statistic.
pvalue: Wald test p-value.
padj: Benjamini–Hochberg adjusted p-value (FDR).
Number of rows: 14455 genes.

Supplemental movies

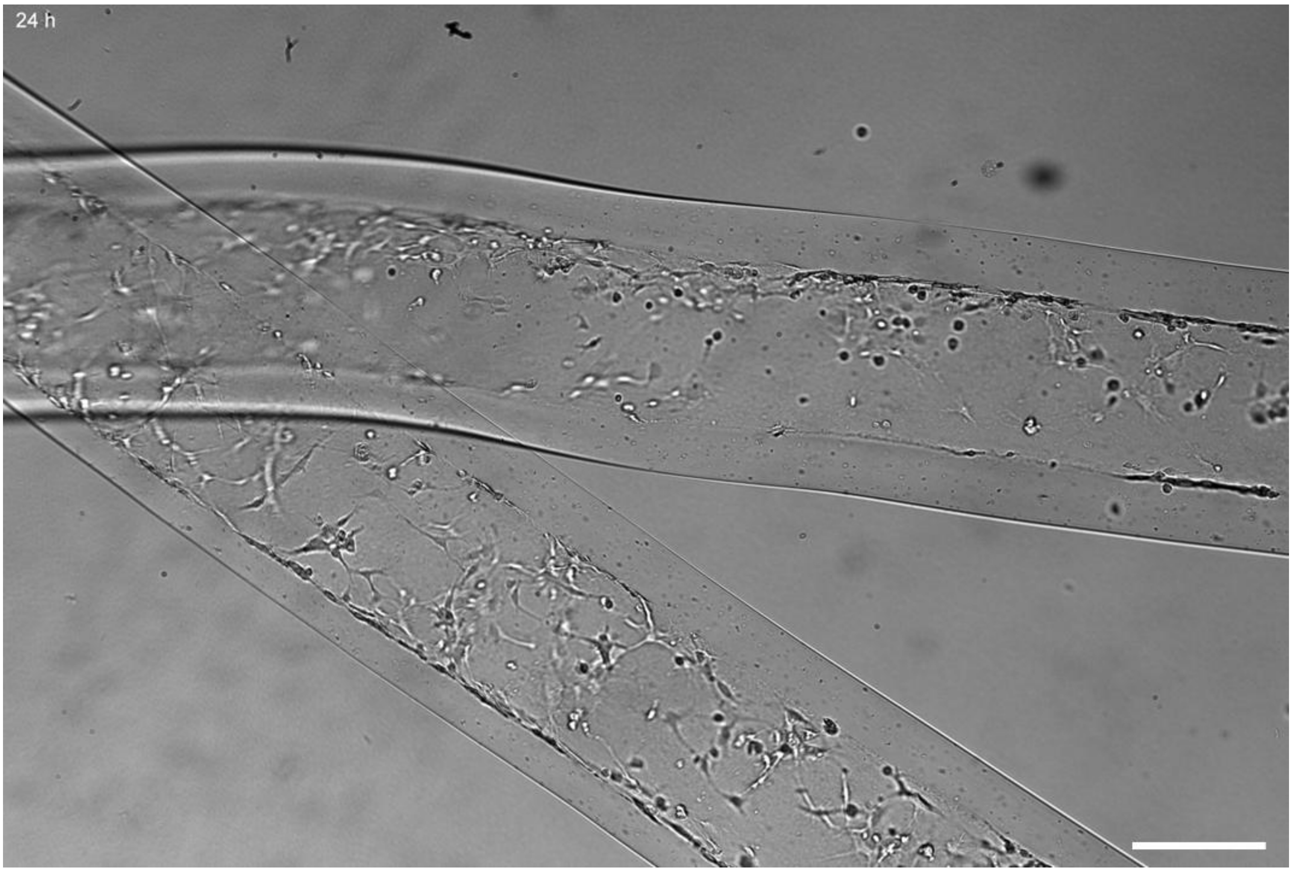

Supplementary Movie S1. Time-lapse monitoring of HDLEC organization and gelatin remodeling in four-component matrices (G_{rh}/MG/HA/FG) containing rhodamine-labeled gelatin (G_{rh})
Brightfield time-lapse sequence showing the dynamic behavior of HDLECs embedded within the hydrogel core. One image was acquired every 3 hours using an Incubascope system (see [10]). Progressive lymphatic endothelial reorganization is observed throughout a 28-day culture period.
Scale bar: 100 µm.

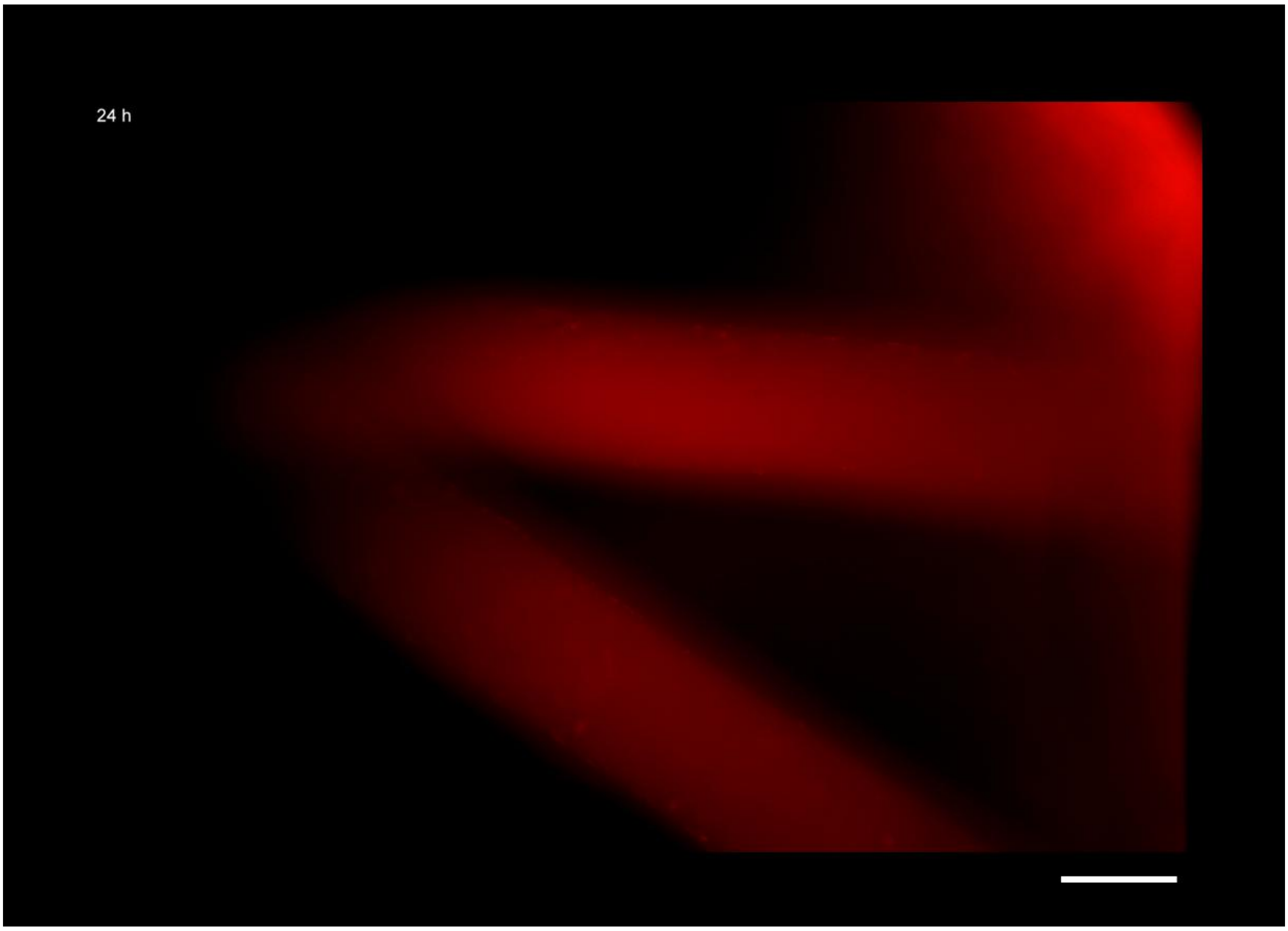

Supplementary Movie S2. Time-lapse monitoring of gelatin remodeling in four-component matrices seeded with HDLECs

Live fluorescence imaging from day 1 to day 29 showing rhodamine-labeled gelatin (G_{rh}, red) within a G_{rh}/MG/HA/FG matrix seeded with HDLECs. One image was acquired every 3 hours using an Incubascope system (see [10]). Progressive loss of G_{rh} signal becomes apparent around day 13, consistent with gelatin degradation and matrix remodeling by embedded cells.

Scale bar: 100 µm.

References

[1] Yue K, Trujillo-de Santiago G, Alvarez MM, Tamayol A, Annabi N, Khademhosseini A. Synthesis, properties, and biomedical applications of gelatin methacryloyl (GelMA) hydrogels. Biomaterials 2015; 73: 254–271. https://doi.org/10.1016/j.biomaterials.2015.08.045

[2] Benton G, Kleinman HK, George J, Arnaoutova I. Multiple uses of basement membrane-like matrix (BME/Matrigel) in vitro and in vivo with cancer cells. Int J Cancer 2011; 128(8): 1751–1757. https://doi.org/10.1002/ijc.25781

[3] Alessandri K, Feyeux M, Gurchenkov B, Delgado C, Trushko A, Krause K-H, et al. A 3D printed microfluidic device for production of functionalized hydrogel microcapsules for culture and differentiation of human neuronal stem cells (hNSC). Lab Chip 2016; 16(9): 1593–1604. https://doi.org/10.1039/c6lc00133e

[4] Andrique L, Recher G, Alessandri K, Pujol N, Feyeux M, Bon P, et al. A model of guided cell self-organization for rapid and spontaneous formation of functional vessels. Sci Adv 2019; 5(6): eaau6562. https://doi.org/10.1126/sciadv.aau6562

[5] Saha S, Fan F, Alderfer L, Graham F, Hall E, Hanjaya-Putra D. Synthetic hyaluronic acid coating preserves the phenotypes of lymphatic endothelial cells. Biomater Sci 2023; 11(22): 7346–7357. https://doi.org/10.1039/d3bm00873h

[6] Knezevic L, Schaupper M, Mühleder S, Schimek K, Hasenberg T, Marx U, et al. Engineering Blood and Lymphatic Microvascular Networks in Fibrin Matrices. Front Bioeng Biotechnol 2017; 5: 25. https://doi.org/10.3389/fbioe.2017.00025

[7] Alderfer L, Saha S, Fan F, et al. Multi-parameter tunable synthetic matrix for engineering lymphatic vessels. Commun Biol 2024; 7: 1262. https://doi.org/10.1038/s42003-024-06935-7

[8] Barnett SN, Cujba A-M, Yang L, et al. An organotypic atlas of human vascular cells. *Nat Med* 2024; 30: 3468–3481. https://doi.org/10.1038/s41591-024-03376-x

[9] Mobed-Miremadi M, Djomehri S, Keralapura M, Schaub N. Fickian-Based Empirical Approach for Diffusivity Determination in Hollow Alginate-Based Microfibers Using 2D Fluorescence Microscopy and Comparison with Theoretical Predictions. Materials 2014; 7(12): 7670–7688. https://doi.org/10.3390/ma7127670

[10] Badon A, Andrique L, Mombereau A, Rivet L, Boyreau A, Nassoy P, et al. The Incubascope: a simple, compact and large field of view microscope for long-term imaging inside an incubator. R Soc Open Sci 2022; 9(2): 211444. https://doi.org/10.1098/rsos.211444
